# ARID1A regulates DNA repair through chromatin organization and its deficiency triggers DNA damage-mediated anti-tumor immune response

**DOI:** 10.1093/nar/gkae233

**Published:** 2024-04-08

**Authors:** Ali Bakr, Giuditta Della Corte, Olivera Veselinov, Simge Kelekçi, Mei-Ju May Chen, Yu-Yu Lin, Gianluca Sigismondo, Marika Iacovone, Alice Cross, Rabail Syed, Yunhee Jeong, Etienne Sollier, Chun- Shan Liu, Pavlo Lutsik, Jeroen Krijgsveld, Dieter Weichenhan, Christoph Plass, Odilia Popanda, Peter Schmezer

**Affiliations:** Division of Cancer Epigenomics, German Cancer Research Center (DKFZ), INF280, 69120 Heidelberg, Germany; Division of Cancer Epigenomics, German Cancer Research Center (DKFZ), INF280, 69120 Heidelberg, Germany; Division of Cancer Epigenomics, German Cancer Research Center (DKFZ), INF280, 69120 Heidelberg, Germany; Division of Cancer Epigenomics, German Cancer Research Center (DKFZ), INF280, 69120 Heidelberg, Germany; Division of Cancer Epigenomics, German Cancer Research Center (DKFZ), INF280, 69120 Heidelberg, Germany; Division of Cancer Epigenomics, German Cancer Research Center (DKFZ), INF280, 69120 Heidelberg, Germany; Division of Proteomics of Stem Cells and Cancer, German Cancer Research Center (DKFZ), INF581, 69120 Heidelberg, Germany; Division of Cancer Epigenomics, German Cancer Research Center (DKFZ), INF280, 69120 Heidelberg, Germany; Division of Cancer Epigenomics, German Cancer Research Center (DKFZ), INF280, 69120 Heidelberg, Germany; Division of Cancer Epigenomics, German Cancer Research Center (DKFZ), INF280, 69120 Heidelberg, Germany; Division of Cancer Epigenomics, German Cancer Research Center (DKFZ), INF280, 69120 Heidelberg, Germany; Division of Cancer Epigenomics, German Cancer Research Center (DKFZ), INF280, 69120 Heidelberg, Germany; Division of Cancer Epigenomics, German Cancer Research Center (DKFZ), INF280, 69120 Heidelberg, Germany; Division of Cancer Epigenomics, German Cancer Research Center (DKFZ), INF280, 69120 Heidelberg, Germany; Division of Proteomics of Stem Cells and Cancer, German Cancer Research Center (DKFZ), INF581, 69120 Heidelberg, Germany; Heidelberg University, Medical Faculty, Heidelberg, Germany; Division of Cancer Epigenomics, German Cancer Research Center (DKFZ), INF280, 69120 Heidelberg, Germany; Division of Cancer Epigenomics, German Cancer Research Center (DKFZ), INF280, 69120 Heidelberg, Germany; German Cancer Consortium (DKTK), INF280, 69120 Heidelberg, Germany; Division of Cancer Epigenomics, German Cancer Research Center (DKFZ), INF280, 69120 Heidelberg, Germany; Division of Cancer Epigenomics, German Cancer Research Center (DKFZ), INF280, 69120 Heidelberg, Germany

## Abstract

AT-rich interaction domain protein 1A (ARID1A), a SWI/SNF chromatin remodeling complex subunit, is frequently mutated across various cancer entities. Loss of ARID1A leads to DNA repair defects. Here, we show that ARID1A plays epigenetic roles to promote both DNA double-strand breaks (DSBs) repair pathways, non-homologous end-joining (NHEJ) and homologous recombination (HR). ARID1A is accumulated at DSBs after DNA damage and regulates chromatin loops formation by recruiting RAD21 and CTCF to DSBs. Simultaneously, ARID1A facilitates transcription silencing at DSBs in transcriptionally active chromatin by recruiting HDAC1 and RSF1 to control the distribution of activating histone marks, chromatin accessibility, and eviction of RNAPII. ARID1A depletion resulted in enhanced accumulation of micronuclei, activation of cGAS-STING pathway, and an increased expression of immunomodulatory cytokines upon ionizing radiation. Furthermore, low ARID1A expression in cancer patients receiving radiotherapy was associated with higher infiltration of several immune cells. The high mutation rate of ARID1A in various cancer types highlights its clinical relevance as a promising biomarker that correlates with the level of immune regulatory cytokines and estimates the levels of tumor-infiltrating immune cells, which can predict the response to the combination of radio- and immunotherapy.

## Graphical Abstract

**Graphical Abstract:**
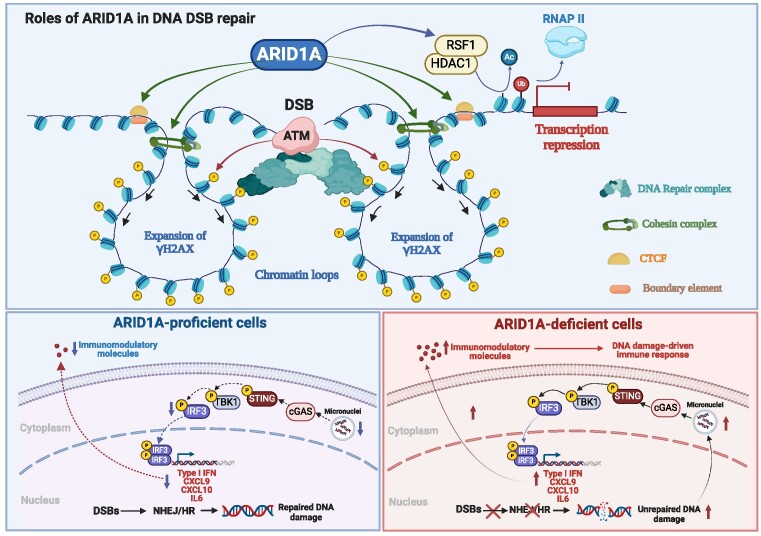

## Introduction

Double-strand breaks (DSBs) are widely acknowledged as the most genotoxic form of DNA damage (1). The DNA damage response (DDR) signaling pathway is activated in order to promote recruitment and activation of the key players involved in repair. DSBs are repaired via two main pathways, non-homologous end-joining (NHEJ) and homologous recombination (HR). (2,3). The induction of DSBs stimulates several changes in the chromatin environment, mediated by epigenetic enzymes catalyzing the modifications of several histones. This results in spreading and amplifying the damage signals as well as promoting the recruitment of DNA repair complexes at the DSBs (4–10). Several chromatin remodeling factors such as Switch/sucrose non-fermentable complex (SWI/SNF), Imitation SWItch complex (ISWI), PARP1, CHD2 and INO80 complex were reported to mediate histone modifications and nucleosome disassembly leading to chromatin relaxation to facilitate DNA repair (11–16). SWI/SNF chromatin-remodeling complexes, also known as BRG1/BRM-associated factor (BAF) complexes, regulate nucleosome positioning in an ATP-dependent manner (17,18). Three subfamilies of SWI/SNF complexes exist in mammalian cells: canonical BAF (cBAF) (19,20), polybromo-associated BAF (PBAF) (21,22), and non-canonical BAF (ncBAF) (23–25). The three complexes share common core subunits, including SMARCC1, SMARCC2, SMARCD1, and one of the two ATPases SMARCA4 or SMARCA2 (referred to as BRG1 or BRM, respectively). The distinctive subunits unique to each of the complexes confer their individual identities (26,27).

The AT-rich interaction domain protein 1A (ARID1A) or (BAF250a) is the largest subunit of the SWI/SNF complex (cBAF complex) and has a DNA-binding domain. ARID1A has a high loss of function mutation rate in many cancers (27–30). ARID1A mutations can lead to cancer phenotypes as shown with colon cancer in mice (31), which suggests a tumor suppressive function of ARID1A. Several reports showed that ARID1A, along with the ATPase SWI/SNF subunit SMARCA4 (BRG1), is recruited to DSBs to facilitate DNA repair (32–34). In which DSB repair pathway ARID1A is involved, remains controversial. While a study reported that ARID1A deletion leads to delayed recruitment of NHEJ proteins and a decrease in NHEJ activity (35), another study demonstrated that ARID1A is recruited to enable end processing to produce RPA-coated single-stranded DNA, thus maintaining ATR activation and HR activity (36).

Despite the growing body of evidence, showing that ARID1A promotes DSB repair, it is not yet fully understood by which mechanisms ARID1A is involved in the regulation of each DSB repair pathway, in particular, NHEJ and HR. Moreover, ARID1A is a well-known chromatin remodeler which contributes to the chromatin organization, but this role has not yet been investigated in the context of DSB repair.

DNA repair deficiency and genomic instability leads to accumulation of cytosolic DNA and formation of micronuclei. Subsequently, this promotes the activation of the cytosolic DNA sensing cyclic GMP-AMP synthase/stimulator of interferon genes (cGAS/STING) signaling pathway in cancer cells (37). This leads to the phosphorylation of TANK-binding kinase 1 (TBK1) and interferon regulatory factor 3 (IRF3), induces a type I interferon response and, thus, produces immunomodulatory cytokines and chemokines (38,39). These molecules enhance the migration of immune cells in cancer, particularly macrophages, and regulate anti-tumor immune response (40,41). Furthermore, the immune checkpoint inhibitors' (ICI) therapeutic effects were reported to be dependent on the cGAS/STING pathway (42,43). Given the high mutation rate of ARID1A in several cancers, it is of clinical importance to know whether ARID1A loss correlates with the accumulation of unrepaired DNA damage, the level of immune regulatory cytokines, and the levels of tumor-infiltrating immune cells. This will help to stratify the cancer patients and define who will benefit from a combination of DNA damaging therapy and immunotherapy, which is still unmet medical need.

Here, we show that ARID1A is a potential epigenetic regulator of both DSB repair pathways, NHEJ and HR. It regulates the formation chromatin loops necessary for efficient γH2AX *foci* formation at DSB sites. Simultaneously, ARID1A is involved in the regulation of transcription repression at DSBs within active transcribed regions. ARID1A loss promotes the accumulation of micronuclei, activation of cGAS-STING pathway and an increased expression of immunomodulatory cytokines and chemokines upon ionizing radiation (IR) treatment. The high mutation rate of ARID1A in various cancer types highlights its clinical relevance as a promising biomarker for further therapeutic studies.

## Materials and methods

### Key resources

#### Reagents

Protease inhibitor cocktail (Roche Diagnostics, Cat#11836170001). Phosphatase inhibitor cocktail (Roche Diagnostics, Cat#04906837001). Vectashield Antifade mounting medium (Vector Laboratories, Cat#H-1000). BisBenzamide H33342 trihydrochloride (Sigma, Cat#B2261). Crystal violet (Merck, Cat# C-0775). Puromycin (Merck, Cat# P8833). Geneticin disulfate (G418) (Roth, Cat#2039.3). Penicillin-Streptomycin (Sigma, Cat#P0781). Novex ECL HRP Chemiluminescent Kit (Invitrogen, Cat#WP20005). High sensitivity HRP Chemiluminescent Kit (Merck, Cat#WBKLS0500). PVDF membrane (Thermo Fisher, Cat#88520). Magna ChIP Protein A magnetic beads (Merck, Cat#16-661). ChIP-Grade Protein G magnetic beads (Cell Signaling, Cat#9006S). Agencourt AMPure XP (Beckman Coulter, Cat#A63880). SuperScript™ III First-Strand Synthesis (Thermofisher, Cat#18080-051). PrimaQUANT™ SYBR green kit (Steinbrenner Laborsysteme, Cat#SL-9902). MinElute PCR Purification kit (Qiagen, Cat#28006). Lipofectamine DharmaFECT 1 (Dharmacoon, Cat# T-2001-03). TransIT-LT1 (Mirus Bio, Cat#MIR 2300). pCBASceI (Addgene, Cat#26477). Q5 high-fidelity DNA polymerase (NEB, Cat#M0491S). Neon™ Transfection System (Thermo Fisher, Cat#MPK10025). Alt-R® CRISPR-Cas9 crRNA (IDT technologies). Alt-R® CRISPR-Cas9 tracrRNA (IDT technologies). Tagment DNA TDE1 Enzyme and Buffer Kit (Illumina, Cat# 20034198). Agilent High Sensitivity D1000 ScreenTape Agilent High Sensitivity D1000 Reagents (Agilent, Cat#5067-5585).

#### Antibodies

Rabbit-anti-ARID1A (Cell Signaling, Cat#12354), Rabbit-anti-ARID1A (Abcam, Cat#ab182560). Mouse-anti-beta-Actin (Santa Cruz, Cat#sc-47778). Mouse-anti-Vinculin (Santa Cruz, Cat#sc-25336). Mouse-anti-phospho-Histone H2A.X (S139) (Merck, Cat#05-636). Rabbit-anti-phospho-Histone H2A.X (S139) (Abcam, Cat# ab2893). Rabbit-anti-XRCC4 (Abcam, Cat#ab213729). Rabbit-anti-BRCA1 (Abcam, Cat#ab9141). Rabbit-anti-RAD21 (Abcam, Cat#ab992). Mouse-anti-Histone H2B (Abcam, Cat#ab52484). Rabbit-anti-CtIP (Abcam, Cat#70163). Rabbit-anti-CTCF (Abcam, Cat#ab128873). Rabbit-anti-HDAC1 (Abcam, Cat#ab1767). Rabbit-anti-pS2-PolII (Abcam, Cat#ab5095). Rabbit-anti-SA1 (Abcam, Cat#ab4457). Rabbit-anti-IRF3 (Abcam, Cat#ab76409). Rabbit-anti-pS386-IRF3 (Abcam, Cat#ab76493). Rabbit-anti-H3K27ac (Abcam, Cat#ab4729). Rabbit-anti-H2AK118ac (PTM BIO, Cat#PTM-173). Rabbit-anti-H2AK119ub (Cell Signaling, Cat#8240S). Mouse-anti-Flag-HRP (Sigma, Cat#A8592). Mouse-anti-IgG (Santa Cruz, Cat#sc-2025). Goat-anti-mouse IgG-HRP (Cell Signaling, Cat#7076P2). Goat-anti-rabbit IgG-HRP (Cell Signaling, Cat#7074S). Goat-anti-mouse IgG-AlexaFluor 594 (Molecular Probes, Cat#A11005). Goat-anti-rabbit IgG-AlexaFluor 488 (Molecular Probes, Cat#A11008). Mouse -anti-dsDNA (Abcam, Cat#ab27156). Rabbit -anti-cGAS (Cell Signaling, Cat#79978S)

#### Laboratory instruments

FACSCanto™ II Flow Cytometer (BD, Cat#338960). Agilent 4150 TapeStation system (Agilent, Cat#G2992AA). Amersham Imager 680 (GE Healthcare, Cat#29270769). Axioplan 2 imaging microscope (Zeiss). LightCycler® 480 (Roche, Cat#05015243001). microTUBE AFA Fiber Pre-Slit Snap-Cap (Covaris, Cat#80606). M220 Focused-ultrasonicator (Covaris). Gammacell® 40 Exactor (Theatronics). Thermocycler (Eppendorf).

#### Biological resources

AID-DIvA cells (originally U2OS cells integrated with AsiSI- expressing vector) were cultured as in (44,45). Upon addition of 4-hydroxytamoxifen to the culture medium, the AsiSI enzyme is localized to the nucleus and generates several DSBs in the genome. U2OS-EJ5 and U2OS-DR were cultured as in (46). MDA-MB-231 (ATCC Cat# HTB-26, RRID:CVCL_0062) were cultured in Dulbecco's modified Eagle's medium (DMEM) supplemented with 10% (vol/vol) fetal bovine serum (BioChrom), 100 U/ml penicillin, 100 μg/ml streptomycin (Sigma-Aldrich). Cells were maintained in a humidified incubator with an atmosphere of 5% CO2 at 37°C. AID-DIvA cells were kindly provided by Dr G. Legube (University of Toulouse, France) and U2OS-EJ5 and U2OS-DR were kindly provided by Dr G. Stark (City of Hope center, USA). Cells were routinely tested to be mycoplasma-free. Competent *E*. *coli* DH5α were used for transformations.

### siRNA and plasmid transfection

A set of four siGENOME upgrade siRNAs were obtained from Dharmacoon (Supplementary Table S1), pooled together and transfected using Lipofectamine DharmaFECT1 (Dharmacon) according to the manufacturer's protocol. Plasmid transfections were carried out using TransITLT1 (Mirus Bio) according to the manufacturer's protocol. Plasmids were transfected 48h after siRNA treatment.

### Generation of ARID1A-knockout cells

Two separate CRISPR guide RNA sequences (crRNA#1: 5ʹ-TATGGGTTAGTCCCGCCATA-3ʹ and crRNA#2: 5ʹ-CGGTACCCGATGACCATGCA-3ʹ) targeting the exon1 of ARID1A open reading frame were designed using IDT Alt-R® CRISPR-Cas9 guide RNA design tool and were ordered as Alt-R® CRISPR-Cas9 crRNA (IDT technologies). Annealing of crRNA and Alt-R® CRISPR-Cas9 tracrRNA (IDT technologies), electroporation of the Cas9:crRNA:tracrRNA complex to the cells and knockout clones were established and confirmed by Western blotting and Sanger sequencing as described previously (47).

### Whole-cell protein extracts and western blotting

Whole-cell protein extraction and Western blotting were performed as previously described (47).

### DNA-damage induction using IR and chemical agents

Irradiation was done with the GammaCell 40 from Theatronics. Wild-type (WT) or ARID1A-KO AID-DIvA cells were treated with 300 nM 4-hydroxytamoxifen (4OHT) for 4 h to induce AsiSI introgression into the nucleus and generation of DSBs. DSB induction was terminated by Auxin supply as described previously (44). The type of repair pathway acting at the different DSB sites had been described previously (44).

### Clonogenic survival assay, DSB repair reporter assay, Immunofluorescence analysis and extraction of chromatin fractions

Assays were performed as previously described (47).

### Chromatin immunoprecipitation and qPCR (ChIP-qPCR)

Chip-qPCR was performed as described previously (47). DSB induction and termination were done as described above. Cells were cross-linked with formaldehyde (1%) and cell pellets were obtained and washed in ice-cold PBS. Nuclei were isolated and then transferred into Adaptive Focused Acoustics (AFA) for chromatin shearing. 25 μg of the sheared chromatin was incubated with 2–5 μg of antibody overnight at 4°C. Pre-blocked magnetic beads were added to the antibody-chromatin mixture and incubated for 3 h at 4°C on a rotator. Beads were washed and incubated with an Elution buffer supplemented with proteinase K, then treated with RNAse and stored at 4°C. DNA was then eluted and purified using Ampure beads (Agencourt AMPure XP) at room temperature (with a ratio 1:1.4). Then washed twice with 80% Ethanol, dried, and finally eluted in dH_2_O at room temperature. For qPCR, proximal (80 bp) or distal (800 bp) primers for the indicated DSBs or primers from the promoters of the indicated DNA repair genes were used (Supplementary Table S1). The qPCR was performed using primaQUANT™ SYBR green kit.

### In-suspension breaks labeling in situ and sequencing (sBLISS)

sBLISS was done as described previously (48). DSBs were induced and induction terminated in WT and ARID1A-KO (AID-DIvA) cells (*n* = 2 biological replicates) as described above. DSB induction was terminated by Auxin and cells were collected at the indicated time points, cross-linked with paraformaldehyde solution and lysed. The nuclei were then permeabilized and non-blunt DSBs were blunted in situ. sBLISS adaptors (Supplementary Table S1) were then ligated to the blunted DSBs in an overnight ligation step. Afterwards, DNA was extracted, purified, and sonicated. The sonicated sBLISS templates were subsequently used as input for in vitro transcription (IVT), in which T7 polymerase specifically amplifies the DSB ends ligated to the sBLISS adaptors, starting from the T7 promoter. Next, the RA3 adaptors (Illumina) (Supplementary Table S1) were ligated to the generated amplified RNA, and with the help of the reverse transcription primer (RTP) (Supplementary Table S1), the RNA molecules were reverse transcribed. Libraries were amplified and indexed in a PCR step that involves one common primer (RP1) and one library-specific indexed primer (RPIX) (Supplementary Table S1). The libraries were purified using AMPure XP beads and measured by Qubit. Fragment sizes were determined by Tape station analysis. Libraries were pooled and subjected to 75-bp single-end sequencing on a Nextseq550 platform at DKFZ Next Generation Sequencing Core Facility.

#### BLISS data analysis

We utilized cutadapt v1.18 for demultiplexing the BLISS data. Subsequently, the datasets from different time points were aligned to the human reference genome (hg19/GRCh37) using bowtie2 (v 2.3.5.1). For peak calling, we employed MACS2 (v 2.1.2.1). The normalization and visualization were performed using Diffbind (v 3.4.11), and all the analyses were conducted in R (v4.1). The genomic regions of all analyzed DSBs (Supplementary Table S2) were tested and validated previously (44,45)

### Circular chromosome conformation capture and sequencing (4C-seq)

4C–seq was done as described previously (49) using WT and ARID1A-KO (AID-DIvA) cells (*n* = 4 replicates) with and without DSBs induced as described above. DSB induction was terminated by Auxin and cells were collected at the indicated time points, cross-linked with 2% formaldehyde for 10 min at room temperature and lysed. Genomic DNA was digested with increasing amounts of *Mbo*I (New England Biolabs, Cat# R0147L) for three rounds of 4 h each followed by ligation with T4 DNA ligase (New England Biolabs, Cat# M0202L). The chromatin was decrosslinked and the DNA purified. A second digestion step was performed with *Nla*III overnight (New England Biolabs, Cat# R0125L) followed by a second ligation step. DNA was purified and measured by Qubit. Five ng of template DNA was PCR-amplified using the viewpoint primers (Supplementary Table S1). The PCR products were purified by AMPure XP beads and measured by Qubit. 4C-seq libraries were generated under qPCR conditions using 2 ng DNA from the previous PCR and Illumina adaptor sequences including a unique index for each condition. DNA libraries were purified by AMPure XP beads and measured by Qubit. Fragment sizes were determined by Tape station analysis. Libraries were pooled and subjected to 75 bp paired-end sequencing on a Nextseq550 platform at DKFZ Next Generation Sequencing Core Facility. 4C-seq data processing and analysis was done according to (50) with pipe4C using single reads starting with the MboI or the NlaIII restriction site; the pipe4C pipeline was applied with default parameters under R3.6.2.

#### 4C-seq data analysis

4C-seq downstream data analysis was performed using the DeepTools2 Web server (51). https://deeptools.readthedocs.io/en/develop/content/about.html). Comparison of two bam files, the tool bamCompare was utilized. The following parameters were used -operation log_2_, - bin size 50, - normalization: Bins Per Million mapped reads (BPM), -smoothLength 10 000. To generate coverage plots, the tool bamCoverage was employed. The parameters used were: - bin size 50, -normalization: Bins Per Million mapped reads (BPM, -smoothLength 10 000). In boxplots, the box represents the interquartile range (IQR), the horizontal line stands for the median and the whiskers are positioned at the minimum and maximum values.

### Nuclear lysate extraction and immunoprecipitation (IP)

Extraction of nuclear lysates and IP were performed as previously described (47). Adherent cells were washed with cold PBS, harvested, and pelleted. Cells were lysed in (10 mM HEPES, pH 7.5, 1.5 mM MgCl_2_, 10 mM KCl) supplemented by 1× protease/phosphatase inhibitor and snap-frozen in liquid nitrogen. 1% NP-40 was added to the solution after thawing, and centrifuged at 1500 × g for 15 min at 4◦C. The supernatant (cytosolic lysate) was collected. The pellet (nuclei) was washed with cell lysis buffer and centrifuge again at the same settings. The pellet (nuclei) was lysed in (300 mM NaCl, 20 mM HEPES, pH 7.5, 3 mM MgCl_2_, 20 mMKCl), supplemented by benzonase (DNase), and incubated 30 min at RT. nuclear lysates were obtained by centrifugation at the highest speed for 15 min at 4◦C. The protein concentration was measured using BCA test, then 1.5–2 mg of nuclear lysate were used for IP, mixed with antibody (anti-ARID1A or anti-IgG) and incubated overnight at 4◦C with rotation. In parallel, prewashed proteinA/G magnetic beads were also separately pre-blocked overnight with 0.1% BSA in PBS. On the next day, the pre-blocked beads were added to the lysate–antibody mix and incubated 3 h at 4°C with rotation. Beads were put on magnetic rack and washed three times with TBST followed by a wash with PBS. Beads were either subjected to preparation for mass spectrometry or resuspended in Laemlli buffer (supplemented with 10% -mercaptoethanol) and incubated 10 min at 95°C then loaded on SDS gel.

### Mass spectrometry sample preparation and data acquisition

Samples for mass spectrometry were analyzed as previously described (47). In brief, magnetic beads were then conditioned and subjected to reduction and alkylation. The reaction was quenched with DTT and proteins were digested on beads with a Trypsin/LysC mix (Promega, V5071) at 37°C for 16 h. Digested peptides were desalted with SP3 para-magnetic beads (52–54). Peptides were eluted, loaded on a trap column and separated over a 25 cm analytical column using the Thermo Easy nLC 1200 nanospray source (Thermo EasynLC 1200, Thermo Fisher Scientific). Peptides were analyzed on a Tri-Hybrid Orbitrap Fusion mass spectrometer (Thermo Fisher Scientific) operated in positive (+2 kV) data-dependent acquisition mode with HCD fragmentation. The MS1 and MS2 scans were acquired in the Orbitrap and ion trap, respectively with a total cycle time of 3 s. MS1 detection occurred at 120 000 resolution, AGC target 1E6, maximal injection time 50 ms and a scan range of 375–1500 *m*/*z*. Peptides with charge states 2 to 4 were selected for fragmentation with an exclusion duration of 40 s. MS2 occurred with CE 33%, detection in topN mode and scan rate was set to Rapid. AGC target was 1E4 and maximal injection time allowed of 50 ms. Data were recorded in centroid mode.

#### Mass spectrometry data processing, analysis and visualization

RAW data were processed with Maxquant software (v 1.5.1.2) including the Andromeda search engine (55,56). Peptide identification was performed as previously described (47). FDR was set to 1% at both protein and peptide levels. Match between runs option was enabled, Label-Free Quantification (LFQ) and iBAQ calculated. For further protein analysis, Perseus free software was used (57). Two-sided t-test statistics were used for the generation of the volcano plots based on LFQ log_10_ values of expressed proteins. FDR was 0.05 and S0 constant was 0.1. Using anti-IgG as control, we set a cutoff for Log2fold changes >2. Pathway enrichment analysis was done using the Metascape resource (58).

### Assay for transposase-accessible chromatin using sequencing (ATAC-seq)

ATAC-seq and its analysis were performed as previously described (47) using WT and ARID1A-KO (AID-DIvA) cells (*n* = 3 replicates each) with and without DSBs induced as described above. DSB induction was terminated by Auxin. Subsequently, nuclei were isolated and resuspended in a 2× transposition buffer and the tagmentation reaction was performed by adding the Tagment DNA Enzyme 1 (Illumina). The mixture was incubated for 30 min at 37°C with mixing at 1000 rpm. The reaction was stopped by 5 M guanidinium thiocyanate (Sigma-Aldrich) and DNA was further purified using AMPure XP beads. ATAC-seq libraries were prepared as previously described (59). The libraries were purified with AMPure XP beads. The concentration of the library was determined using the Qubit dsDNA HS Assay Kit (Thermo Fisher Scientific). Quality control was performed on a Tape station using the Agilent High Sensitivity DNA Kit. Sequencing was performed at the DKFZ Genomics Core Facility using the Nextseq 550 Paired-End 75 bp.

#### ATAC-seq data analysis

Sequencing reads were processed using the CWL-based ATAC-seq workflow (60). Then, IDR analysis (v 2.0.3) was conducted to identify reproducible peaks across biological replicates, applying an IDR threshold of ≤0.05. Differential accessibility analysis was carried out using Diffbind (v 3.4.11), enabling the detection of regions with statistically significant differential accessibility between conditions or groups. All visualization and statistical analyses in R provided insights into the biological relevance of differentially accessible double-strand break regions.

### Mapping of histone modifications by antibody‐guided chromatin tagmentation and sequencing (ACT-seq)

ACT-seq and its analysis was done as described previously using WT and ARID1A-KO (AID-DIvA cells) (*n* = 3 replicates each) with and without DSB induction (61–63). Briefly, *E. coli* (C3013, New England Biolabs) was transformed with plasmid pET15bpATnp (#121137, Addgene), the pA‐Tn5ase protein was isolated. The pA‐Tn5ase was mixed with Tn5ME‐A + B load adaptor mix in a complex formation buffer (CB) to form the pA‐Tn5 transposome (pATn5ome). For every reaction, the pA‐Tn5ome‐antibody (pATn5ome‐ab) complex was formed by mixing 1 μl pA‐Tn5ome with 0.8 μl CB and 0.8 μl antibody solution. Approximately 50 000 nuclei were used for pA‐Tn5ome‐ab complex binding and tagmentation. Tagmented DNA was purified with a MinElute kit (#28004, Qiagen) and eluted with 20 μl elution buffer (EB). Sequencing libraries were generated under real‐time conditions with a LightCycler 480 in a reaction mix consisted of tagmented DNA eluate, NEBNext High Fidelity 2X Mix, 100X SYBRGreen, primer Tn5McP1n, and Custom Nextera PCR Barcode. Libraries were purified with AMPure XP beads with a bead:DNA ratio of 1.4:1. Quantity and fragment size of the libraries were determined with a Qubit dsDNA HS assay kit and a TapeStation 4150 with D1000 High Sensitivity Assay, respectively. Samples were multiplexed and sequenced on NextSeq 550 system (paired‐end, 75 bp) with mid‐output at the DKFZ Genomics Core Facility.

#### ACT-seq data analysis

We employed the nf-core CUTANDRUN pipeline (https://nf-co.re/cutandrun/3.2) to analyze our ACT-seq data with BPM (Base Pair Mappability) normalization. This pipeline handled critical tasks, including adapter trimming, Bowtie2-based read alignment to the human reference genome (hg19/GRCh37), and the peak calling was done using SEACR. The called peak regions and BPM-normalized read counts generated by the pipeline were then used for the subsequent comparisons and visualization through deepTools (v 3.5.1).

### RNA isolation and gene expression analysis by real-time qPCR

RNA extraction, reverse transcription and RT-qPCR using intron-spanning primers (Supplementary Table S1) were performed as described previously (47). Data is presented as the mean ± SD value in independently repeated experiments.

### RNA sequencing

Sequencing libraries were prepared by the Genomics and Proteomics Core Facility (DKFZ, Heidelberg) from total RNA using the Illumina TrueSeq Stranded Total RNA Library Prep Kit according to the manufacturer's instructions. Samples were sequenced in a paired-end setting (100 bp) on an Illumina NovaSeq 6000 machine for sequencing.

#### RNAseq data analysis

Data was processed by the Omics IT and Data Management Core Facility (DKFZ, Heidelberg), using the Roddy RNA-seq Workflow. Default parameters were used unless mentioned otherwise. Sequences were aligned to the human reference genome (hg19/GRCh37) by applying the software STAR (64). Gene counts were generated with featureCounts (65) and the gene annotation v.29 lift 37. For the identification of differentially expressed genes, the R library DESeq2 was used (66). Closest TSS and genes to the DSBs were determined through Rsubread library (v 2.14.2). Genes with an adjusted p-value < 0.05 were defined as significantly differentially expressed genes (DEG).

### Translocation assay

Assay was performed as described previously (67). Briefly, AID-DIvA cells were treated as mentioned above and DSB induction was terminated by Auxin. DNA was extracted using the DNAeasy kit (Qiagen). Improper rejoining frequencies between the DSBs were assessed by qPCR using specific primers (Supplementary Table S1). The DSBs are as the followings: two HR-prone DSBs located at *MIS12* and *TRIM37* (chr17_5390209 and chr17_57184285), two NHEJ-prone DSBs *LINC00217* and *LYRM2* (chr6_135819337 and chr6_9034817), or two inter-chromosomal HR-prone DSBs at *TRIM37* and *RBMXL1* (chr17_57184285 and chr1_89433139). Results were normalized using two control regions, both far from any AsiSI sites and γH2AX domain. Delta-Delta-Ct method was used to calculate the normalized translocation frequencies.

### Micronuclei, cytosolic dsDNA and cGAS staining

Experiments were performed as described previously (68). Briefly, Cells were seeded on slides, incubated overnight and then irradiated with 4 Gy. Cells were collected 24 h post IR, fixed in 4% paraformaldehyde, mildly permeabilized in 0.01% Triton, blocked, and finally incubated overnight at 4°C with primary antibodies against cytosolic dsDNA and cGAS. Then, slides were washed in PBS, briefly blocked, and incubated for 45 min at RT with secondary antibody. Nuclear DNA is stained using bisBenzimide (Sigma Aldrich) in Tris–HCl. The mounting medium Fluoromount-G (Southern Biotech, Cat# 0100-01) was added before sealing with a cover slide. Fluorescent images were taken by using the Nikon ND2 imaging microscope. Discrete DNA accumulations separated from the primary nucleus were defined as micronuclei in cells with normal nuclear morphology, all apoptotic cells were excluded. The overlap between micronuclei, cytosolic dsDNA and cGAS was monitored.

### Correlation of ARID1A expression with cytokines expression and immune cells infiltration using TCGA data of cancer patients

Gene expression and clinical data were downloaded from TCGA using TCGAbiolinks (69). Samples were separated into four groups in each cancer type by applying the *k*-means clustering algorithm to ARID1A gene expression values. Only group 1 and 4 where the median ARID1A expression value is lowest and highest was used. xcell was used for estimating immune infiltration in each sample (70). Un-stranded transcripts per million (TPM) values were used for the gene expression profile due to the requirement of xcell. The radiation treatment information was acquired from the clinical data.

### Statistical analyses

Unless stated, GraphPad Prism v5 software was used to create graphs, perform statistical tests, and calculate p-values. Statistical analyses for BLISS, 4C-seq, ATAC-seq and ACT-seq were performed using R version 3.6 (71). The graphical abstract was created with BioRender.com.

## Results

### ARID1A is required for both DSB repair pathways, NHEJ and HR

**Figure 1.**
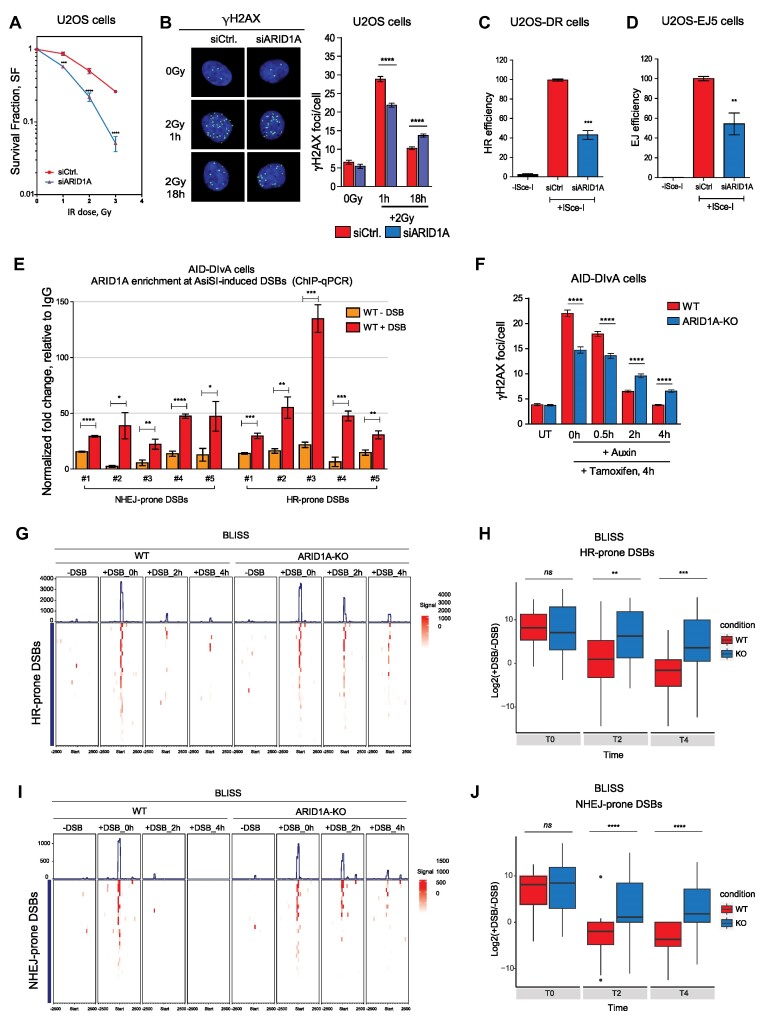
ARID1A promotes DSB repair pathways. (**A**) Clonogenic survival assay of U2OS cells transfected either with control siRNA (siCTR) or a pool of four siRNAs targeting ARID1A (siARID1A) and treated with the indicated dose of ionizing radiation. Data are presented as mean ± SEM, One-Way ANOVA with Bonferroni's multiple comparison test was performed. (**B**) Representative micrographs and quantification of IR-induced γH2AX *foci* in U2OS cells, ca. 500 cells were counted at indicated time points. Data are presented as mean ± SEM, One-Way ANOVA with Bonferroni's multiple comparison test was performed. (**C**, **D**) HR efficiency measured in U2OS-DR cells and NHEJ efficiency measured in U2OS-EJ5 cells using the indicated siRNAs, respectively. Data are presented as mean ± SD, one-way ANOVA with Tukey's multiple comparison test ws performed. (**E**) Enrichment of ARID1A at the indicated DSBs in WT AID-DIvA cells, measured by ChIP-qPCR. Data are presented as mean ± SEM, Student's *t* test was performed. (**F**) Quantification of ASiSI-induced γH2AX foci in AID-DIvA cells, ca. 500 cells were counted at indicated time points. Data are presented as mean ± SEM, one-way ANOVA with Bonferroni's multiple comparison test was performed. (**G**) Tornado plots showing BLISS signals in WT and ARID1A-KO cells at HR-prone DSBs at the indicated time points. (**H**) Quantification of BLISS signals in WT and ARID1A-KO cells at HR-prone DSBs at the indicated time points. (**I**) Tornado plots showing BLISS signals in WT and ARID1A-KO cells at NHEJ-prone DSBs at the indicated time points. (**J**) Quantification of BLISS signals in WT and ARID1A-KO cells at NHEJ-prone DSBs at the indicated time points. All data presented in this figure are from *n* = 3 independent experiments (biological replicates). Statistical significance is presented as: * P< 0.05,** P < 0.01, *** P < 0.001, **** P < 0.0001, ns = not significant.

To confirm the previously reported role for ARID1A in DNA DSB repair in our cell models, we performed knockdown of ARID1A in U2OS cells (Supplementary Figures S1A–C), followed by IR and analyzed the survival and the number of γH2AX *foci*. ARID1A-depleted cells showed higher cellular sensitivity to IR as compared to control cells (Figure 1A). This was accompanied by fewer numbers of induced γH2AX *foci* 1 h after treatment, and a higher number of residual γH2AX *foci* 18 h after treatment (Figure 1B). The efficiency of both HR and NHEJ was assessed by DSB reporters in U2OS-DR and U2OS-EJ5 cells, respectively. Knockdown of ARID1A decreased the efficiency of both DSB pathways (Figures 1C and D), confirming a deficiency in DNA DSB repair. We examined chromatin recruitment of pathway-specific DNA repair proteins that act within HR (BRCA1 and CtIP) and NHEJ (KU70 and XRCC4) after IR in control and ARID1A-depleted cells. Western blot analyses of the chromatin-bound fractions of ARID1A-depleted cells showed reduced accumulation of BRCA1, CtIP, KU70 and XRCC4, at an early time point after IR (Supplementary Figures S1D). This was not due to reduction of the overall expression of those DNA repair proteins upon ARID1A depletion (Supplementary Figure S1E). Together, these results confirm a role for ARID1A in DNA DSB repair.

IR can induce diverse DNA lesions in addition to DSBs. AID-DIvA cells (44) were used to study the accumulation of ARID1A at DSBs. ChIP-qPCR revealed the accumulation of ARID1A at NHEJ- and HR-prone DSBs after DNA damage induction, supporting a role of ARID1A in both DNA DSB repair mechanisms (Figure 1E). Using ARID1A knockout AID-DIvA cells (ARID1A-KO cells; Supplementary Figure S1F), we assessed DNA DSB repair by counting γH2AX *foci*. We observed significantly less induced γH2AX *foci* immediately after DSB induction and a higher number of persistent γH2AX *foci* 2h and 4h after induction (Figure 1F), confirming defective γH2AX *foci* formation and resolution. A delay in the DSB repair process and accumulation of persistent unrepaired HR-prone and NHEJ-prone DSBs in ARID1A-KO cells were observed (Figures 1G–J). The reduction in γH2AX *foci* formation at an early time point was not due to lower number of induced DSBs in ARID1A-KO cells, as BLISS labeling demonstrated comparable induction of DSBs to WT cells. ARID1A is a well-known chromatin remodeler, and it may contribute to a proper chromatin structure to facilitate γH2AX *foci* formation and accumulation of repair complexes of both repair pathways. Collectively, our data suggests an upstream or epigenetic role of ARID1A in DSB repair.

### ARID1A promotes the formation of chromatin loops at DSBs in response to DNA damage

**Figure 2.**
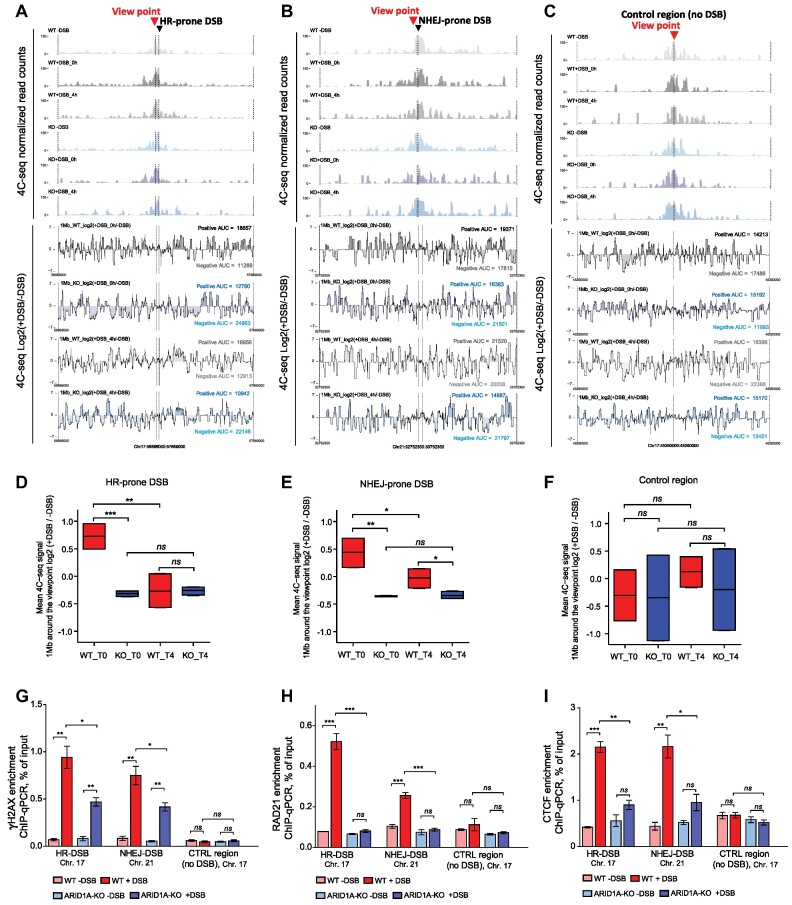
ARID1A is required for efficient chromatin loop formation. (**A**–**C**) 4C–seq normalized read counts at indicated viewpoints as well as differential 4C–seq track (log_2_ +DSB/–DSB) in WT (grey) ARID1A-KO (blue) cells collected at indicated time points (T0 and T4). 4C–seq data were smoothed using 10-kb spans. (**D**–**F**) Box plots showing the differential 4C–seq (log_2_ +DSB/–DSB) at indicated viewpoints. Four technical replicates from two independent experiments (biological replicates), data are presented mean ± SD, Student's *t* test. (**G**–**I**) Enrichment of γH2AX, RAD21 and CTCF, respectively, at the indicated DSBs in WT and ARID1A-KO cells, measured by ChIP-qPCR. *n* = 3 independent experiments (biological replicates); data are presented as mean ± SEM, Student's *t* test. Statistical significance is presented as: * P < 0.05, ** *P* < 0.01, *** *P* < 0.001, **** *P* < 0.0001, ns = not significant.

Chromatin loop formation has been described to contribute to γH2AX deposition suggesting that the spread of γH2AX *foci* is controlled by chromosome architecture (72,73). Loss of ARID1A was associated with the weakening of the chromatin loop and remodeling of the topologically associated domains (TADs) in hepatocytes (74). Here we asked whether ARID1A is also involved in chromatin loops formation at DSBs to facilitate the establishment of γH2AX *foci*. ARID1A-KO and WT AID-DIvA cells were collected before and after induction of DSBs, and 4C-seq was performed to monitor the interaction between the damaged genomic regions and its surrounding loci, such interaction reflects the chromatin loop formation needed for γH2AX deposition. In WT cells, we observed an increased interaction between viewpoints and surrounding loci at both HR-prone and NHEJ-prone DSBs upon DSB induction (T0), while this was not the case at the control undamaged region (Figures 2A–C). The interactions at DSBs declined and reached comparable levels as seen in the undamaged control region after 4 h (T4). Depletion of ARID1A strongly impaired the overall increase in interaction between the DSBs and their adjacent regions at T0, and showing further drop at T4 (Figures 2A–C). By comparing ARID1A-KO to WT cells, significantly lower interactions at the DSBs were observed in ARID1A-KO cells at T0, while no difference was detected at the control undamaged region (Figures 2D–F). ARID1A constitutes the DNA-binding subunit of the SWI/SNF complex, and its loss can affect the stability of SWI/SNF complex (26). In addition, the perturbation of BRG1, the core ATPase subunit of SWI/SNF complex, showed reduced induction of γH2AX following IR and cellular sensitivity to several DNA damaging agents (75), which is similar to the effects upon ARID1A loss in our hand. We examined whether inhibition of BRG1, the core ATPase subunit of the SWI/SNF complex, can also mimic the effect of ARID1A depletion on chromatin loop formation. By treating the WT cells with BRG1 ATPase inhibitor (BRM014) (76), the interactions between the DSBs and their adjacent regions at T0 were reduced, but after release from the inhibitor these interactions were recovered (Supplementary Figures S2A and B). Inhibition of BRG1 ATPase activity led to reduced interactions at the HR-prone DSB at T0 to a level similar to ARID1A-KO, while interactions at NHEJ-prone DSB were partially reduced and no difference was detected at the control undamaged region (Supplementary Figures S2C–E). Collectively, our data indicates that the ability of DSBs to interact with adjacent loci, i.e. to form a chromatin loop within the damaged TAD, requires the presence of both ARID1A and the ATPase activity of the SWI/SNF complex subunits BRG1.

### ARID1A regulates the recruitment of RAD21 and CTCF to maintain the TADs

TAD and chromatin loop formation on both sides of DSBs depend on binding of the cohesin complex component SCC1/RAD21 and the CTCF insulator protein to DSBs (73,77). We performed ChIP-qPCR to test the accumulation of γH2AX, RAD21 and CTCF at the DSBs in our 4C viewpoints as well as other representative HR- and NHEJ-prone DSBs, which have been tested and validated previously (44,47). Consistent to our results, depletion of ARID1A significantly reduced γH2AX enrichment at HR - and NHEJ-prone DSBs (Figure 2G). We observed enhanced accumulation of RAD21 and CTCF at both NHEJ- and HR-prone DSBs after induction of DSBs in WT cells, whereas the depletion of ARID1A significantly impaired this accumulation at the tested DSBs (Figures 2H and I, and S2F and G). This indicates that loss of ARID1A impairs the recruitment of RAD21 and CTCF, potentially leading to weakening or loss of chromatin loops and TADs in the damaged chromatin, which subsequently prevent proper formation of γH2AX. Our proteomic data analysis (using a significance threshold for an FDR <0.05, and a cut-off for log_2_ fold change > 2) revealed 1074 protein interactions with ARID1A (Supplementary Table S3). A strong enrichment was observed for pathways associated with chromatin remodeling, cell cycle, chromosome organization, DNA metabolic process, epigenetic regulation of gene expression and DNA repair (Supplementary Figure S2H). Several DSB repair related-factors were identified (Supplementary Table S4), which are directly or indirectly involved in DNA repair. This indicates that ARID1A can be involved directly in DNA repair process via promoting the recruitment of DNA repair factors, as reported previously in several studies (35,36). Following our observations regarding the accumulation of RAD21 and CTCF at DSBs, both were identified among the proteins interacting with ARID1A (Supplementary Figure S2I). This was further confirmed by co-immunoprecipitation experiments, as we observed enhanced interaction between ARID1A and RAD21 after DNA damage (Supplementary Figure S2J). Together our data indicates that ARID1A binds to RAD21 and CTCF and promotes their recruitment to regulate the chromatin organization into TADs and formation of chromatin loops which are required for establishment of γH2AX *foci* at damaged loci. Our results suggest that ARID1A regulates both HR and NHEJ pathways via facilitating the formation of chromatin loops at the DSBs.

### ARID1A regulates chromatin accessibility at DSBs

**Figure 3.**
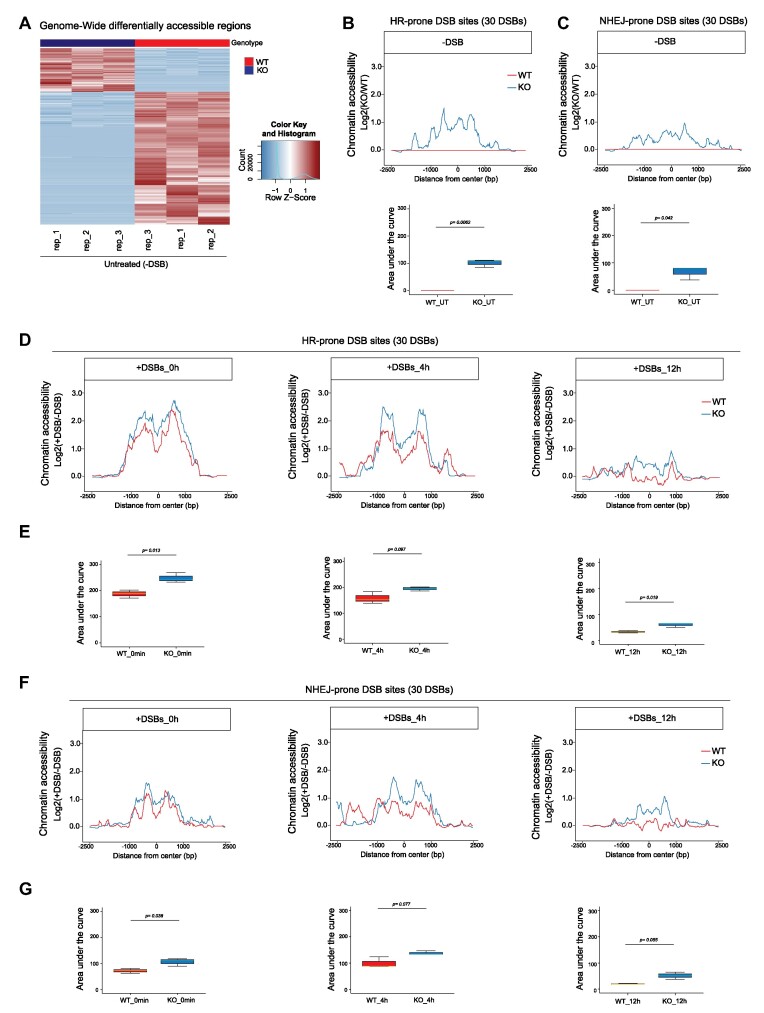
ARID1A loss alters chromatin accessibility at DSBs. (**A**) Heatmap showing the genome-wide differentially accessible region in ARID1A-KO, measured by ATAC-seq. (**B**, **C**) Profile plot showing the differentially chromatin accessibility in ARID1A-KO (upper panel), as well as box plot showing the differential accessibility (lower panel, at HR-prone and NHEJ-prone DSBs, respectively. data are presented as mean ± SD, and Student's *t* test was performed. (**D**) Profile plots showing the differentially chromatin accessibility in WT and ARID1A-KO cells at HR-prone and at indicated time points after DSBs induction. (**E**) Box plots showing the differential accessibility, at HR-prone DSBs, data are presented as mean ± SD, and Student's *t* test was performed. (**F**) Profile plots showing the differentially chromatin accessibility in WT and ARID1A-KO cells at NHEJ-prone and at indicated time points after DSBs induction. (**G**) Box plots showing the differential accessibility, at NHEJ-prone DSBs, Student's *t* test was used. All data presented in this figure are from *n* = 3 independent experiments (biological replicates).

Since ARID1A contributes to the regulation of chromatin accessibility to enhancers (78) and DSBs (35), we mapped open chromatin sites in WT and ARID1A-KO cells. We found 90 210 differentially accessible regions (DARs) in ARID1A-KO cells. The majority displayed loss of chromatin accessibility (63 755 sites, 70.7%) (Figure 3A). We found that 40% of the accessibility gaining regions were enriched in or close to promoters, while the accessibility loosing regions were enriched in introns or distal regions (Supplementary Figure S3A). Without DSB induction, chromatin accessibility at both HR- and NHEJ-prone DSBs was higher in ARID1A-KO cells compared to WT (Figures 3B and C). This can be explained by the genomic distribution of the DSB regions, as more than 75% were enriched in or close to promoters (Supplementary Figure S3A), so the majority of the DSBs were among the accessibility gaining regions. After induction, WT cells showed generally an increased chromatin accessibility around the center of the HR-prone DSBs at T0, with a slight drop close to DSBs. This DNA damage-induced accessibility declines gradually with time, which overlaps with the ongoing DNA repair kinetics, until it recovers completely after 12 h (Figure 3D). ARID1A-depleted cells displayed a higher accessibility around the HR-prone DSBs compared to WT, which persists even after 12 h (Figures 3D and E). This suggests a role for ARID1A in regulation of chromatin accessibility at HR-prone DSBs after DNA damage induction. Similar to ARID1A depletion, the inhibition of BRG1 ATPase activity showed a higher chromatin accessibility around the HR-prone DSBs compared to WT (Supplementary Figures S3B and C). At NHEJ-prone DSBs, both WT and ARID1A-KO cells showed induction of chromatin accessibility following DNA damage, however, to a lesser extent compared to HR-prone DSBs, possibly because HR-prone DSBs are close to actively transcribed genes, while NHEJ-prone DSBs are not. At NHEJ-prone DSBs, chromatin accessibility was higher in ARID1A-KO than in WT cells at T0 and after 12 h (Figures 3F and G). Similar results were also obtained upon BRG1 ATPase inhibitor treatment (Supplementary Figures S3D and E). These results suggest that the loss of ARID1A or BRG1 ATPase function significantly alters the chromatin accessibility before and after induction of DSBs.

### ARID1A interacts with the HDAC1-RSF1 complex to control the distribution of activating histone marks at DSBs

**Figure 4.**
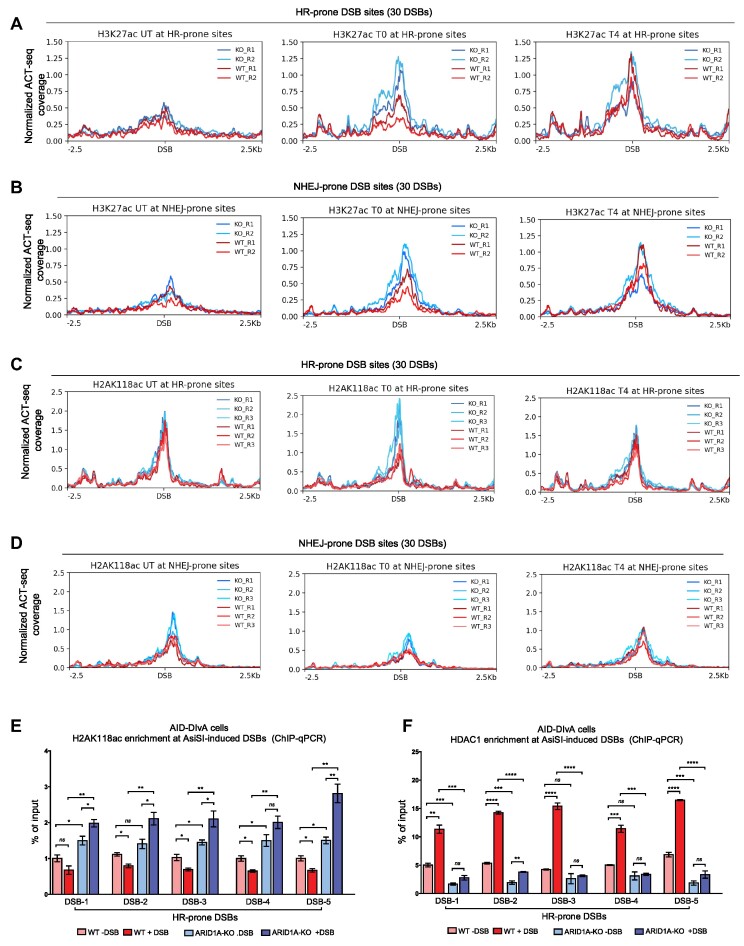
ARID1A interacts with the HDAC1-RSF1 complex to control the distribution of activating histone marks at the DSBs (**A**, **B**) Normalized ACT-seq coverage showing the enrichment of H3K27ac at HR- and NHEJ-prone DSBs, respectively, at the indicated time points in WT and ARID1A-KO cells. (**C**, **D**) Normalized ACT-seq coverage showing the enrichment of H2A118ac at HR- and NHEJ-prone DSBs, respectively, at the indicated time points in WT and ARID1A-KO cells. (**E**, **F**) Enrichment of H2AK118ac and HDAC1, respectively, at the HR-prone DSBs in WT and ARID1A-KO cells, measured by ChIP-qPCR. Data are presented as mean ± SEM, and Student's t test was performed. All data presented in this figure are from *n* = 3 independent experiments (biological replicates). Statistical significance is presented as: * P < 0.05, ** *P* < 0.01, *** *P* < 0.001, **** *P* < 0.0001, ns = not significant.

To explore the underlying mechanism that drives the observed changes in chromatin accessibility, we monitored the distribution of activating histone marks around DSBs. The enrichment of H3K27ac, a marker for enhancers and accessible chromatin, was enhanced upon DNA damage in both WT and ARID1A-KO cells around the HR- and NHEJ-prone DSBs. ARID1A-KO showed higher enrichment of H3K27ac around the DSBs at time point T0 (Figures 4A and B). This is consistent with our corresponding ATAC-seq data (Figures 3D–G). In ARID1A-depleted cells, the higher chromatin accessibility and acetylation of H3K27 can subsequently affect the transcription of the genes within the genomic regions where the DSBs were induced. Since HR-prone DSBs are located close to promoters of actively transcribed genes and are more abundant in transcription-elongation associated histone marks, while NHEJ-prone DSBs are not (44); thus, transcription might be suppressed at HR-prone DSBs during DNA repair. We analyzed the accumulation of histone H2A acetylated at lysine 118 (H2K118ac), known to be enriched in transcriptionally active regions and to regulate transcription suppression during DSB repair (79). After DSB induction (T0), a reduced level of H2AK118ac was observed in WT but not in ARID1A-KO cells; the level recovered later at T4 (Figure 4C). Consistently, similar patterns were also detected at the closest transcription start sites (TSS) (Supplementary Figure S4A). By looking at NHEJ-prone DSBs and their closest TSS sites, we observed a lower overall level of H2AK118ac compared to HR-prone DSBs, confirming that these DSBs are at transcriptionally inactive loci. However, Both WT and KO cells showed a slight decrease in this histone mark after induction of DSBs, with a higher level in the KO cells (Figures 4D and S4B). ChIP-qPCR confirmed the enhanced enrichment of H2AK118ac in KO cells before DSB induction, showing further increase after DSB induction, whereas a decrease in the H2AK118ac level was detected after DSB induction in WT cells (Figure 4E). The HDAC1 complex together with the chromatin remodeler RSF1 induces the de-acetylation of H2AK118ac; this de-acetylation precedes the ubiquitination of histone H2A at lysine 119, which in turn promotes the displacement of the elongating RNAPII and represses the transcription at adjacent TSS (79). ARID1A was reported to interact with HDAC1 and promote HDAC1 recruitment to chromatin (80). In accordance with this, we detected HDAC1 and RSF1 as interaction partners of ARID1A in our proteomic analysis (Supplementary Figure S4C), and this interaction can occur before and after DNA damage (Supplementary Figure S4D). Loss of ARID1A led to decreased recruitment of HDAC1 at HR-prone DSBs before and after DSBs induction (Figure 4F). Altogether, our data suggests that ARID1A is involved in distribution of epigenetic marks around HR-prone DSBs.

### ARID1A is involved in transcription repression near HR-prone DSBs

**Figure 5.**
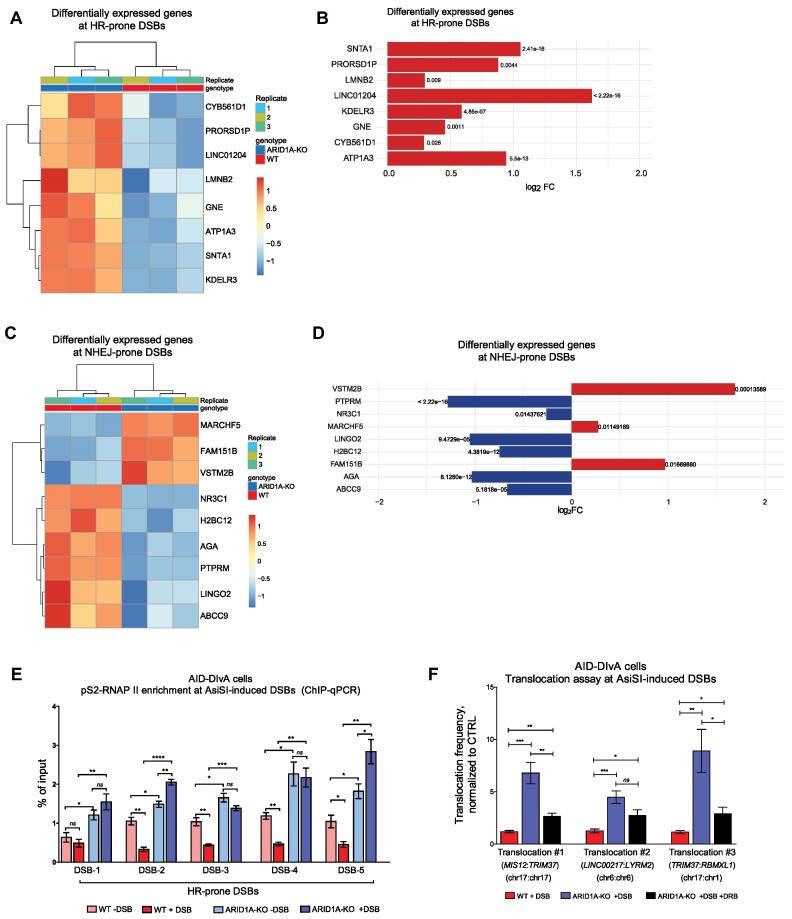
ARID1A regulates the transcription repression at HR-prone DSBs. (**A**) Heat map showing differentially expressed genes that are located in close proximity to HR-prone DSBs. (**B**) Bar plot showing the upregulation of the genes located close to HR-prone DSBs in ARID1A-KO cells. (**C**) Heat map showing differentially expressed genes that are located in close proximity to HR-prone DSBs. (**D**) Bar plot showing the upregulation of the genes located close to NHEJ-prone DSBs in ARID1A-KO cells. (**E**) Enrichment of pS2-RNAPII at the HR-prone DSBs in WT and ARID1A-KO cells, measured by ChIP-qPCR. Data are presented as mean ± SEM, and Student's *t* test was performed. (**F**) Translocation frequencies in WT and ARID1A-KO AID-DIvA cells measured by qPCR of *MIS12:TRIM37*, *LINC00271:LYRM2* and *RIM37:RBMXL1* rejoining after DSB induction, normalized to untreated control. Data are presented as mean ± SEM, and Student's *t* test was performed. All data presented in this figure are from *n* = 3 independent experiments (biological replicates). Statistical significance is presented as: * *P* < 0.05, ** *P* < 0.01, *** *P* < 0.001, **** *P* < 0.0001, ns = not significant.

Higher accumulation of H2AK118ac around TSS sites close to HR- and NHEJ-prone DSBs in ARID1A-KO versus WT cells (Supplementary Figure S4A) indicates higher transcription even before DNA damage induction. RNA-seq analysis showed that genes close to HR-prone DSBs are upregulated in ARID1A-KO cells (Figures 5A and B), while the majority of genes close to NHEJ-prone DSBs were downregulated (Figure 5C and D). Moreover, the transcript per million (TPM) of the listed genes confirmed the upregulation of the genes close to HR-prone DSBs in ARID1A-KO cells (Supplementary Figures S5A and B). This indicates that the deficiency of H2AK118ac de-acetylation in ARID1A-KO cells leads to continuous transcription of genes close to HR-prone DSBs. Consistently, the elongating RNA polymerase II (pS2-RNAPII) was enriched in KO cells prior to DSB induction; enrichment was either further increased after DSB induction or remained unchanged. In contrast, WT cells showed reduced enrichment of pS2-RNAPII following DSBs induction, indicating eviction of the elongating RNAPII and transcription silencing (Figure 5E). These results indicate that ARID1A is involved in the regulation of RNAPII eviction and transcription suppression during DSB repair. Collectively, our results suggest two mechanisms by which ARID1A regulates DSB repair, one is via promoting chromatin loop formation and chromatin organization to establish γH2AX within the damaged TADs, second via promoting transcription suppression during DSB repair to avoid transcriptional errors and genomic instability.

Since ARID1A loss led to DNA repair deficiency, the unrepaired DSBs can be prone for chromosomal translocations. We measured intra- and inter-chromosomal translocations frequency at both HR- and NHEJ-prone DSBs after DNA damage induction. ARID1A-KO cells showed increased intra- and inter-chromosomal translocations frequency at both HR- and NHEJ-prone DSBs (Figure 5F). The inhibition of transcription partially reduced the translocation events only at HR-prone DSBs. This indicates that the defect in transcription suppression in ARID1A-KO cells is not the only mechanism that leads to translocations.

### ARID1A loss leads to activation of the cGAS/STING-mediated immune response.

**Figure 6.**
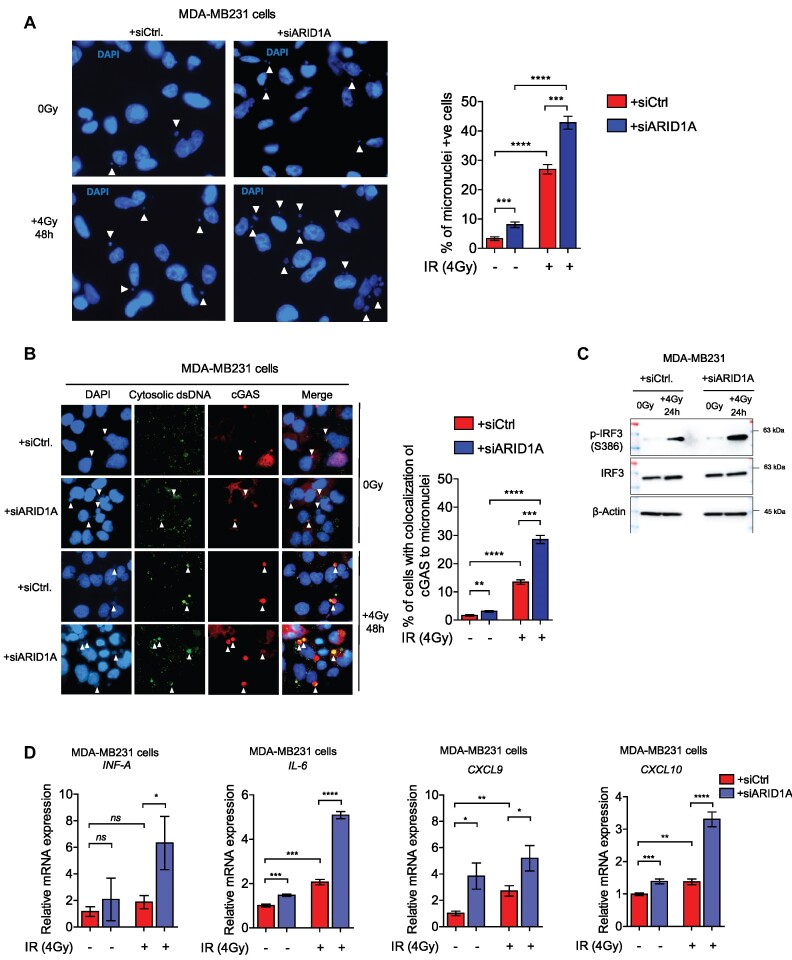
ARID1A depletion is associated with activation of cGAS/STING-mediated immune response. (**A**) Representative micrographs and quantification of micronuclei in MDA-MB231 cells transfected either with control siRNA (siCTR) or a pool of four siRNAs targeting ARID1A (siARID1A) and treated with the indicated dose of ionizing radiation. 500 cells were counted at indicated time points. *n* = 3 independent experiments (biological replicates); data are presented as mean ± SEM, and Student's *t* test was performed. (**B**) Representative micrographs and quantification of the overlap between micronuclei, cytosolic dsDNA and cGAS in MDA-MB231 transfected either with control siRNA (siCTR) or a pool of four siRNAs targeting ARID1A (siARID1A) and treated with the indicated dose of ionizing radiation. 500 cells were counted at indicated time points. *n* = 3 independent experiments (biological replicates); data are presented as mean ± SEM, and Student's t test was performed. (**C**) Western blot expression analysis of p-IRF3 in MDA-MB231 cells transfected either with siCTR or siARID1A and treated with the indicated dose of ionizing radiation. Representative WB from 3 independent experiments (biological replicates). (**D**) mRNA expression analysis of cGAS/STING-mediated immune cytokines, done by RT-qPCR. *n* = 3 independent experiments (biological replicates); data are presented as mean ± SEM, and Student's *t* test was performed. Statistical significance is presented as: * P < 0.05, ** *P* < 0.01, *** *P* < 0.001, **** *P* < 0.0001, ns = not significant.

Radiotherapy of certain tumor entities like breast cancer is routinely applied in the clinic. We induced DNA damage by IR of the ARID1A- depleted breast cancer cell line MDA-MB231 and observed increased accumulation of micronuclei, an indication for impaired DNA damage repair (Figure 6A). Accumulation of micronuclei and any cytosolic double stranded DNA (dsDNA) can be detected by cGAS leading to activation of cGAS/STING signaling pathway. We performed immunofluorescence staining of cytosolic dsDNA and cGAS. The results showed an overlap of cGAS staining with the micronuclei and cytosolic dsDNA after IR treatment, the incidence of this overlap was higher in ARID1A-KO cells (Figure 6B). Moreover, ARID1A-depleted cells displayed higher phosphorylation levels of pSer386-IRF3 (Figure 6C), a marker for activation of the cGAS/STING pathway, which detects abnormal cytosolic dsDNA and translocates to the nucleus to activate the expression of type I interferons (IFNs) (81,82). We observed increased expression of genes *INF-α*, *IL-6*, *CXCL9* and *CXCL10* upon IR treatment in both ARID1A-depleted and control cells, but ARID1A loss led to a higher expression levels (Figure 6D). This suggests that the accumulation of unrepaired DNA damage in ARID1A-depleted cells is associated with the expression level of immune regulatory cytokines.

**Figure 7.**
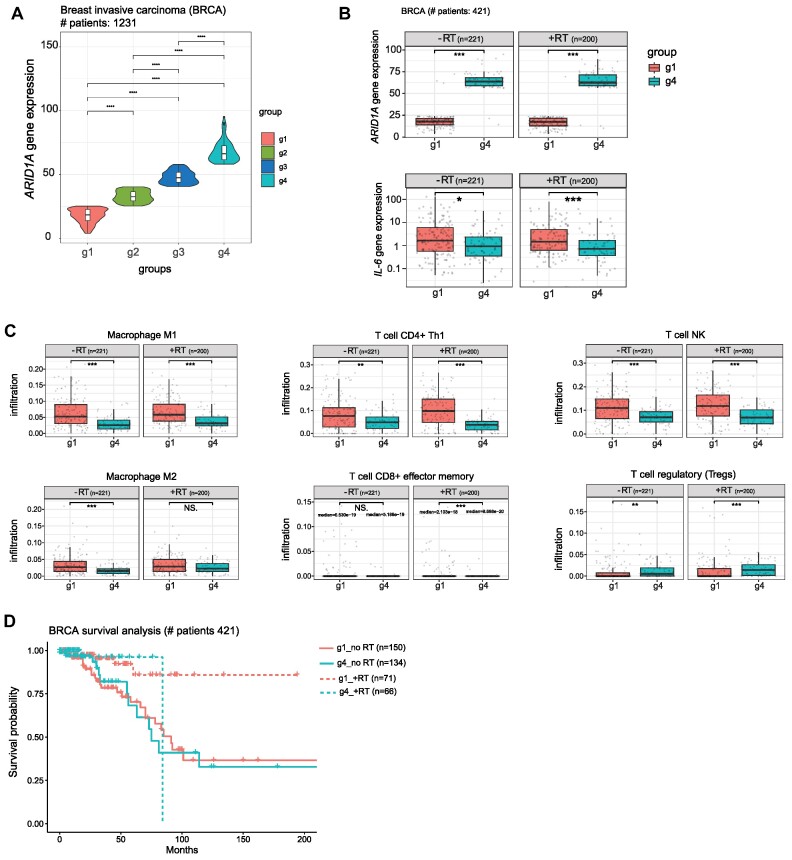
ARID1A depletion is associated with the infiltration of immune cells in cancer patients. (**A**) Box plot representation showing ARID1A expression in clusters of patient samples of TCGA Breast invasive carcinoma (BRCA). (**B**) Box plot showing ARID1A expression in group 1 and 4 of patient samples of TCGA BRCA, who received radiotherapy (upper panel), as well as the expression of IL-6 in the indicated groups (lower panel). (**C**) Box plots showing the level of immune cells infiltration in group 1 and 4 of patient samples of TCGA BRCA, who received radiotherapy. (**D**) Breast cancer patient survival analysis comparing group 1 and 4 with or without radiotherapy treatment. Statistical significance is presented as: * *P* < 0.05, ** *P* < 0.01, *** *P* < 0.001, **** *P* < 0.0001, ns = not significant.

Breast cancer patients with low ARID1A expression, who have received radiotherapy (RT) demonstrated higher *IL-6* expression compared to patients with high ARID1A expression (Figures 7A and B). Activation of the cGAS/STING pathway and production of immunomodulatory molecules such as INF-α, IL-6, CXCL9 and CXCL10 were described to be involved in the enhanced migration of immune cells in cancer, particularly macrophages, and regulate anti-tumor immune response (40,41). Patients with less expressed *ARID1A* exhibited higher infiltration of macrophages M1, myeloid dendritic cells, and CD4+ and CD8+ T cells. On the other hand, less infiltration of immunosuppressive regulatory T cells (Treg) was observed in patient group 1 (Figure 7C). Survival analysis of breast cancer patients showed better survival of patients with low ARID1A expression (group 1) when received RT (Figure 7D). This indicates that ARID1A expression is a promising prognostic marker for patients receiving RT. RT-treated patients with low *ARID1A* expression from other tumor entities, such as skin cutaneous melanoma (SKCM), thyroid carcinoma (THCA), and glioblastoma multiforme (GBM), displayed higher expression of *CXCL10, IL-6* and *CXCL9*. This was accompanied with higher infiltration of macrophages and several other immune cells (Supplementary Figures S6A–F and S7A–C). These results suggest ARID1A expression as a potential biomarker that correlates with the level of immune regulatory cytokines and, hence, might be a proxy for tumor-infiltrating immune cells. Cancer patients with low ARID1A expression would benefit and respond better to the combination of radiotherapy either with DDR inhibitors (DDRi) to increase the load of DNA damage, and/or with immunotherapy to further enhance the functions and the infiltration of immune cells. Such therapeutic combinatorial approaches are promising and need further validation using *in vivo* models.

## Discussion

SWI/SNF complexes are recruited to DSB sites in a manner depends on their interaction with γH2AX (83,84). This is followed by spreading of the γH2AX signals probably by increasing the accumulation of ATM kinase (84). The exact process of how SWI/SNF complexes, particularly ARID1A-containing complexes, expand γH2AX signals was not yet studied. Here, we uncover an epigenetic role of ARID1A in the regulation of γH2AX expansion and DSB repair. Our results suggest that ARID1A and the ATPase active BRG1 are involved in the formation of chromatin loops at DSBs to facilitate γH2AX expansion within the damaged TAD. This leads to the efficient recruitment of key downstream effectors of both NHEJ and HR pathways. TADs are made up of chromatin loops. The formation of chromatin loops is mediated by binding of CTCF to its motifs and by co-localization of CTCF with interacting cohesin (85,86). Our data show that the depletion of ARID1A significantly reduced the accumulation of RAD21 and CTCF at DSBs. Moreover, an interaction between ARID1A and RAD21 has been detected, which was further enhanced following DNA damage. In contrast, a study using hepatocytes without DNA damage induction reported a direct interaction between BRG1 and RAD21, while a similar interaction could not be detected between ARID1A and RAD21(74). We can however expect that both cell type and treatment conditions may define the precise function of SWI/SNF components and their interaction network. In summary, we suggest that the ARID1A-containing SWI/SNF complex regulates the chromatin architecture at DSBs in cooperation with RAD21 and CTCF. RAD21 and CTCF have been reported to regulate γH2AX foci formation (73,87). Loss of cohesin and CTCF resulted in failure of chromatin loop formation and disruption of the boundaries of chromatin loops, respectively (88). CTCF, which acts at chromatin boundaries as an insulator element promoting three-dimensional genome organization, has also been described to facilitate DSB repair via HR (89–92). Different SWI/SNF complexes were associated with chromatin organization into TADs. They interact with architectural elements such as RAD21, CTCF and RNAP II to organize TADs genome-wide (74,93,94).

We also show that ARID1A is required to maintain proper chromatin accessibility at DSBs. Our ATAC-seq results demonstrate that its loss leads to higher chromatin accessibility at DSBs compared to WT cells. In contrast to this, a study has reported that depletion of ARID1A led to a reduction of chromatin accessibility at regions adjacent to a Cas9-induced DSB (35). However, only one DSB was assessed and a different assay was used. In our study, the observed alterations in chromatin accessibility in ARID1A-depleted cells can be caused either directly, by the loss of the chromatin remodeling activity, or indirectly via redistribution of histone post-translational modifications. Since we observed impaired recruitment of RAD21 and CTCF in ARID1A-depleted cells resulting in deregulation of chromatin organization, it may also lead to aberrant spreading of activating histone marks that increases the accessibility of the chromatin region (95). In line with this notion, loss of ARID1A was accompanied by chromatin compartment switching, i.e. from open chromatin compartments to dense chromatin compartments and *vice versa*, which in turn alters transcription (74). Indeed, the activating histone marks H3K27ac and H2AK118ac, which are markers of accessible chromatin and transcription activation, were highly enriched around HR-prone DSBs and to a lower extent at NHEJ-prone DSBs in ARID1A-KO cells following DNA damage induction. This is consistent with our ATAC-seq data showing enhanced chromatin accessibility around the DSBs following DNA damage induction. Under DNA damage, HDAC1 is recruited to DSB sites and induces the de-acetylation of H2AK118ac, a step which is mandatory for the ubiquitination of histone H2A at K119. This in turn promotes the displacement of the elongating RNAPII and leads to transcription repression at TSS sites near the DSBs to ensure accurate DSB repair (79). We detected an interaction between ARID1A and HDAC1, which was present both in the undamaged condition as well as after DNA damage induction. Furthermore, HDAC1 recruitment to DSBs was dependent on ARID1A. In line with our results, ARID1A and BRG1 were shown to interact physically with HDAC1, and ARID1A depletion decreases the DNA binding of BRG1 and HDAC1, which leads to a gain of histone H4 acetylation (80). Consistently, pS2-RNAPII was highly enriched in KO cells prior to DSB induction, which was either further increased after DSB induction or remained unchanged. This suggests that ARID1A is needed for HDAC1-dependent de-acetylation of histones and the subsequent eviction of pS2-RNAPII and transcriptional repression. A recent study has been published demonstrating a role of SWI/SNF complexes in promoting RNAPII eviction, transcription silencing, R-loop resolution, and DNA repair via HR (96). According to their results, ARID1A was not involved initially in the eviction of RNAPII after DNA damage induced by IR, but it is needed to maintain this eviction. The difference between the applied cellular models must however be considered. In our model, we are specifically looking at pure HR-prone DSBs which are induced enzymatically and located in close proximity to actively transcribed genes. Whereas in their model, they used IR-induced DNA damage, which can result in several different DNA lesions beside DSBs. Furthermore, the dissection of the pathway by which the DSBs are repaired is not possible following IR-induced DNA damage.

In summary, we conclude that ARID1A is needed for the establishment of pre-existing chromatin organization and epigenetic marks for DSB repair and transcription silencing. ARID1A regulates both HR and NHEJ pathways via facilitating the formation of chromatin loops at the DSBs. Interestingly, ARID1A contributes to an additional regulatory mechanism for those DSBs in proximity to actively transcribed genes, which is the silencing of transcription during DNA repair. Only HR-prone DSBs are located in actively transcribed genes and are more abundant in transcription-elongation associated histone marks such as H3K36 tri-methylation, which also target these DSBs to HR repair (44). Consistently our results show that HR-prone DSBs exhibit higher chromatin accessibility, H2AK118 acetylation and RNAPII enrichment, which make them more affected by loss of ARID1A than NHEJ-prone DSBs. This indicates that NHEJ-prone DSBs are not regulated by ARID1A via this additional axis. ARID1A depletion resulted in an increased intra- and inter-chromosomal translocation frequency at both HR- and NHEJ-prone DSBs. This high translocation frequency of unrepaired DSBs can be partially rescued by inhibition of transcription but only at HR-prone DSBs. This further confirms that ARID1A plays multiple epigenetic roles in DSB repair, and is consistent with our data that showed defects in chromatin loop formation in ARID1A-depleted cells, which is thought to precede transcription silencing.

DNA repair deficiency and genomic instability lead to accumulation of cytosolic DNA and formation of micronuclei. Subsequently, this can be detected by cGAS leading to activation of cGAS/STING signaling pathway in cancer cells. Several studies reported activation of cGAS/STING signaling pathway either upon DNA damage induced by therapeutic agents (97–99) or upon DSB repair defects due to inactivation of key DSB repair factors such as ATM, ATR, PARP1, RAD51 and BRCA2 (100–104), or upon epigenetic alterations that impact DNA repair such as mutations of histone H3.3 as recently reported by our group (68) and by other research groups as well (105). While inactivation of DNA-PKcs upon irradiation attenuated STAT1 activation, a downstream target of cGAS/STING pathway (106), it enhanced the formation of cytosolic double-stranded DNA and promoted antitumor type 1 IFN signaling in cGAS/STING-independent, but an RNA polymerase III (POL III), retinoic acid-inducible gene I (RIG-I), and antiviral-signaling protein (MAVS)-dependent manner (107). As a result of defective DSB repair in ARID1A depletion, accumulation of micronuclei and activation of cGAS-STING pathway were observed upon IR treatment. This subsequently resulted in an increased expression of several immune-stimulatory cytokines and chemokines, such as INF-α, IL-6, CXCL9 and CXCL10. These molecules are involved in the enhanced migration of immune cells in cancer, particularly macrophages, and regulate anti-tumor immune response (40,41). Consistently, the depletion of PBRM1, a subunit of another SWI/SNF chromatin remodeling complex (the PBAF complex), exhibited enhanced accumulation of micronuclei and activation of cGAS/STING pathway compared to PBRM1-WT cells due to an increased accumulation of unrepaired DNA damage after PARP inhibition (108).

In line with our *in vitro* results, the analysis of TCGA clinical samples demonstrates an increased expression of at least one of the aforementioned immunomodulatory molecules in tumors with low *ARID1A* expression. This was also associated with higher infiltration of macrophages M1, monocytes, myeloid dendritic cells, NK cells, as well as CD4+ and CD8+ T cells. In almost all cancer cases, ARID1A mutations result in loss of ARID1A expression (109), which raises difficulties in making ARID1A therapeutically targetable. It is however of clinical importance to use ARID1A expression as a potential biomarker that correlates with the level of immune regulatory cytokines, and hence can estimate the levels of tumor-infiltrating immune cells. In line with this, it has been reported that tumors lacking ARID1A expression exhibit higher tumor-infiltrating lymphocytes (TILs) and enhanced immune checkpoints, which render these tumors sensitive to immune checkpoint blockade therapy (110,111). Two studies reported an evidence that ARID1A deficiency impacts on immune signalling via another DNA repair mechanisms such as mismatch repair (MMR). Alterations of *ARID1A* were accompanied with mutations in MMR-related genes and associated with higher expression of PD-L1 and better prognosis in patients with endometrial carcinoma. They showed that ARID1A loss can be used as a prognostic marker for immune checkpoint blockade (112,110). This indicates that ARID1A loss can impact immune response to tumor cells via other mechanisms, and it is not restricted only to DSB repair deficiency and activation of cGAS/STING pathway. We also do not exclude any effects that may be mediated by the loss of the epigenetic functions of ARID1A.

One of the most common types of cancer treatment is radiotherapy (RT). Along with its ability to kill or inhibit the growth of cancer cells by increasing the rate of DNA damage, it also has important immune-stimulatory properties, such as upregulation of genes that code for neoantigens and immune-stimulatory cytokines that increase the immunogenicity of the cancer cells (113,114). On the other hand, due to the fractionation schedule of the radiation dose, RT can cause immunosuppression through Hypoxia that induces tumor microenvironment (TME) remodeling. This results in accumulation of immunosuppressive regulatory T cells (Treg), exhausted T cells (Tex), and M2 macrophages (113,114). The combination of radiotherapy either with DDRi and/or immunotherapy in cancer patients with low ARID1A expression is a promising therapeutic approach to improve treatment response of cancer patients. In accordance with our suggestion, an increased tumor-infiltrating lymphocytes and longer patient survival were associated with low expression of both ARID1A and ATM/Chk2 axis, and inhibition of ATM selectively potentiated the efficacy of immune checkpoint blockade in ARID1A-depleted tumors but not in WT tumors (115). Therefore, an *in vivo* screening of several DDRi combined with Radio- and immunotherapy in ARID1A-depleted tumors will have a translational impact as it will provide potential therapeutic approaches to improve cancer treatment.

## Supplementary Material

gkae233_Supplemental_Files

## Data Availability

The data that support the findings of this study are presented in the main text paper and in the online Supplementary Information file. Further information and requests for resources and reagents should be directed to and will be fulfilled by the corresponding authors, Ali Bakr (a.bakr@dkfz.de). The generated high throughput sequencing data have been deposited to Gene Expression Omnibus (GEO) under the accession the GEO ID GSE249492. The mass spectrometry proteomics data have been deposited to the ProteomeXchange Consortium via the PRIDE [1] partner repository with the dataset identifier PXD047225. The source codes are available in the Supplementary material.
