# Supplementary material for: ARID1A regulates DNA repair through chromatin organization and its deficiency triggers DNA damage-mediated anti-tumor immune response
Source: Nucleic Acids Res. 2024 Apr 8;52(10):5698–719. doi: 10.1093/nar/gkae233 (PMC11162808; doi:10.1093/nar/gkae233)
Supplement: gkae233_Supplemental_Files [file gkae233_supplemental_files.zip › Revised_Suppl. Figures+legends _.pdf]

Supplementary Figures Titles and Legends

Figure S1: ARID1A promotes DSB repair pathways. (A-C) Western blots showing the knockdown of ARID1A in the indicated cell lines. **(D)** Western blot showing recruitment of the indicated DNA repair factors to the chromatin (left panel), and the quantification of the results (right panel). **(E)** Western blots showing the expression of the indicated DNA repair proteins upon ARID1A knockdown. **(F)** Western blots showing the knockdown of ARID1A in AID-DIvA cells. All data presented in this figure are from n=3 independent experiments (biological replicates). Statistical significance is presented as: ** = p<0.05, ** = p<0.01, *** = p<0.001, **** = p<0.0001, ns= not significant*.

Figure S2: ARID1A and BRG1 are required for efficient chromatin loop formation. (A & B) 4C–seq normalized read counts at indicated viewpoints as well as differential 4C–seq track (log2 +DSB/-DSB) in WT (grey) and BRG1 inhibition (orange). 4C–seq data were smoothed using 10-kb spans. **(C-E)** box plots showing the differential 4C–seq track (log2 +DSB/-DSB) at indicated viewpoints. 4 technical replicates from two independent experiments, data are presented mean ±SD, Student's t test. **(F & G)** Enrichment of RAD21 and CTCF, respectively, at the indicated DSBs in WT and ARID1A-KO cells, measured by ChIP-qPCR. n=3 independent experiments (biological replicates); data are presented as mean ±SEM, Student's t test. **(H)** GO pathway analysis of ARID1A protein interactions done by metascape resource. **(I)** Volcano plot showing ARID1A protein interactions, highlighting RAD21 and CTCF as well as SWI/SNF complex subunits. **(J)** Western blots confirming the interaction between ARID1A, and RAD21 and CTCF. All data presented in this figure are from n=3 independent experiments (biological replicates), unless otherwise stated. Statistical significance is presented as ** = p<0.05, ** = p<0.01, *** = p<0.001, **** = p<0.0001, ns= not significant*.

Figure S3: BRG1 inhibition alters chromatin accessibility at DSBs. (A) Annotation of the genomic regions that gain or lose accessibility in ARID1A-KO cells, as well as annotation of all DSB regions. **(B)** Profile plots showing the differentially chromatin accessibility in WT and BRM014 treated cells at HR-prone and at indicated time points after DSBs induction. **(C)** Box plots showing the differential accessibility, at HR-prone DSBs. **(D)** Profile plots showing the differentially chromatin accessibility in WT and BRM014 treated cells at NHEJ-prone and at indicated time points after DSBs induction. **(E)** Box plots showing the differential accessibility, at NHEJ-prone DSBs. All data presented in this figure are from n=3 independent experiments (biological replicates). Data are presented as mean ±SD, Student's t test. Statistical significance is presented as ** = p<0.05, ** = p<0.01, *** = p<0.001, **** = p<0.0001, ns= not significant*.

Figure S4: ARID1A regulates the transcription repression at HR-prone DSBs (A & B) Normalized ACT-seq coverage showing the enrichment of H2A118ac at the closest TSS sites to HR- and NHEJ-prone DSBs, respectively, at the indicated time points in WT and ARID1A-KO cells. **(C)** Volcano plot showing ARID1A protein interactions, highlighting HDAC1 and RSF1 as well as SWI/SNF complex subunits. **(D)** Western blots confirming the interaction between ARID1A, and HDAC1 and RSF1. All data presented in this figure are from n=3 independent experiments (biological replicates).

Figure S5: ARID1A regulates the transcription repression at HR-prone DSBs. (A) Bar plot showing the number of transcript per million (TPM) of the genes located close to HR-prone DSBs in ARID1A-KO cells. **(B)** Bar plot showing the number of transcript per million (TPM) of the genes located close to NHEJ-prone DSBs in ARID1A-KO cells. All data presented in this figure are from n=3 independent experiments (biological replicates). Data are presented as mean ±SD, Student's t test. Statistical significance is presented as ** = p<0.05, ** = p<0.01, *** = p<0.001, **** = p<0.0001, ns= not significant*.

Figure S6: ARID1A depletion is associated with the infiltration of immune cells in cancer patients. (A) Box plot representation showing ARID1A expression in clusters of patient samples of TCGA Skin Cutaneous Melanoma (SKCM). **(B)** Box plot showing ARID1A expression of group 1 and 4 of patient samples of TCGA SKCM, who received radiotherapy (left panel), as well as the expression of CXCL-10 and CXCL-9 in the indicated groups (right panel). **(C)** Box plots showing the level of immune cells infiltration of group 1 and 4 of patient samples of TCGA SKCM, who received radiotherapy. **(D)** Box plot representation showing ARID1A expression in clusters of patient samples of TCGA Thyroid carcinoma (THCA). **(E)** Box plot showing ARID1A expression of group 1 and 4 of patient samples of TCGA THCA, who received radiotherapy (left panel), as well as the expression of CXCL-10, TNF and STING in the indicated groups

(right panel). **(F)** Box plots showing the level of immune cells infiltration in group 1 and 4 of patient samples of TCGA THCA, who received radiotherapy.Statistical significance is presented as: ** = p<0.05, ** = p<0.01, *** = p<0.001, **** = p<0.0001, ns= not significant*.

Figure S7: ARID1A depletion is associated with the infiltration of immune cells in cancer patients. **(A)** Box plot representation showing ARID1A expression in clusters of patient samples of TCGA Glioblastoma multiforme (GBM). **(B)** Box plot showing ARID1A expression in group 1 and 4 of patient samples of TCGA GBM, who received radiotherapy (left panel), as well as the expression of CXCL-10 and IL-6 in the indicated groups (right panel). **(C)** Box plots showing the level of immune cells infiltration of group 1 and 4 of patient samples of TCGA GBM, who received radiotherapy. Statistical significance is presented as: ** = p<0.05, ** = p<0.01, *** = p<0.001, **** = p<0.0001, ns= not significant*.

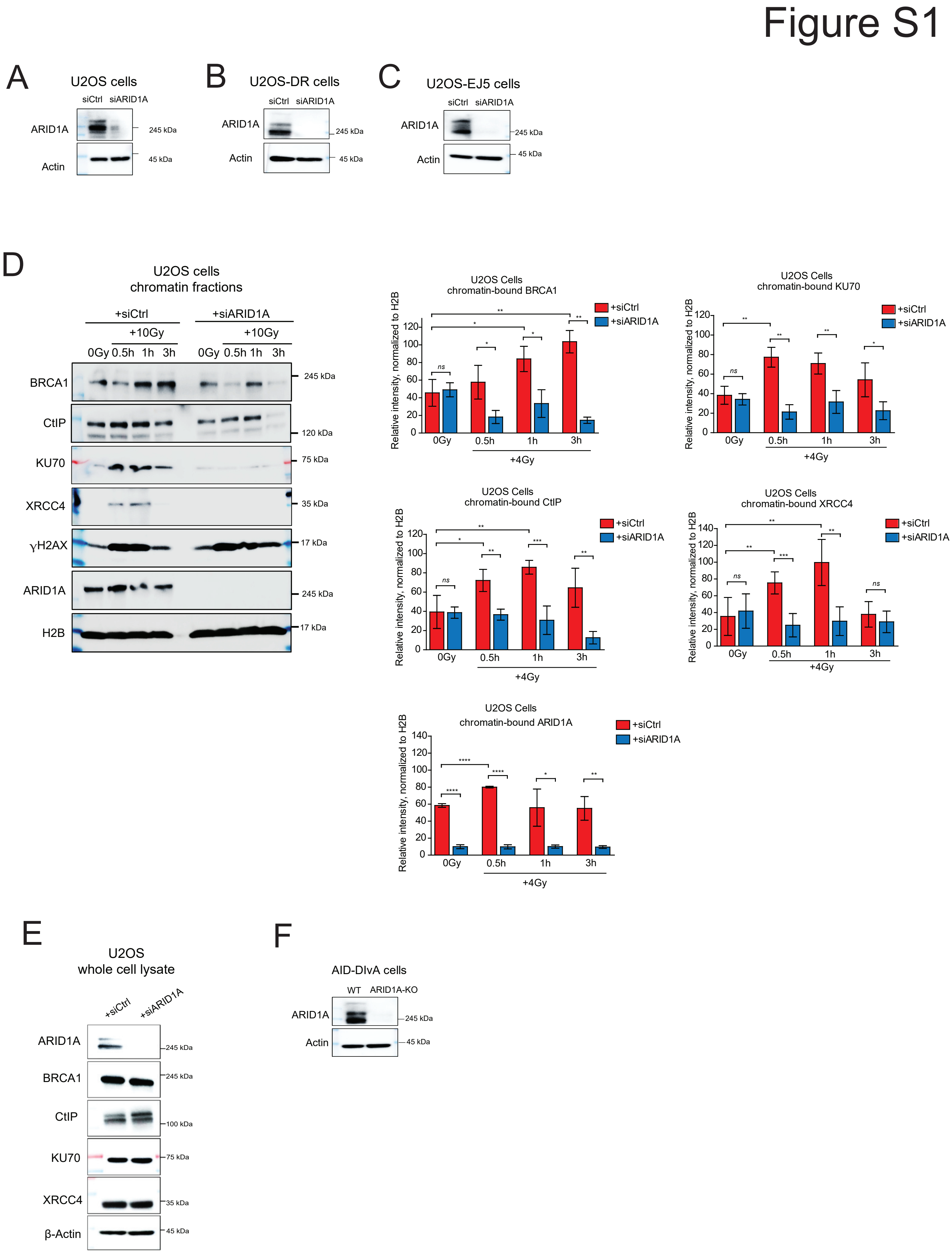

Figure S1
A
U2OS cells
siCtrl
siARID1A
ARID1A
245 kDa
Actin
45 kDa
B
U2OS-DR cells
siCtrl
siARID1A
ARID1A
245 kDa
Actin
45 kDa
C
U2OS-EJ5 cells
siCtrl
siARID1A
ARID1A
245 kDa
Actin
45 kDa
D
U2OS cells
chromatin fractions
+siCtrl
+siARID1A
+10Gy
0Gy 0.5h 1h 3h
BRCA1
245 kDa
CtIP
120 kDa
KU70
75 kDa
XRCC4
35 kDa
γH2AX
17 kDa
ARID1A
245 kDa
H2B
17 kDa
U2OS Cells
chromatin-bound BRCA1
U2OS Cells
chromatin-bound KU70
U2OS Cells
chromatin-bound CtIP
U2OS Cells
chromatin-bound XRCC4
U2OS Cells
chromatin-bound ARID1A
Relative intensity, normalized to H2B
+siCtrl
+siARID1A
0Gy
0.5h
1h
3h
+4Gy
ns
E
U2OS
whole cell lysate
+siCtrl
+siARID1A
ARID1A
245 kDa
BRCA1
245 kDa
CtIP
100 kDa
KU70
75 kDa
XRCC4
35 kDa
β-Actin
45 kDa
F
AID-DIvA cells
WT
ARID1A-KO
ARID1A
245 kDa
Actin
45 kDa

Figure S2

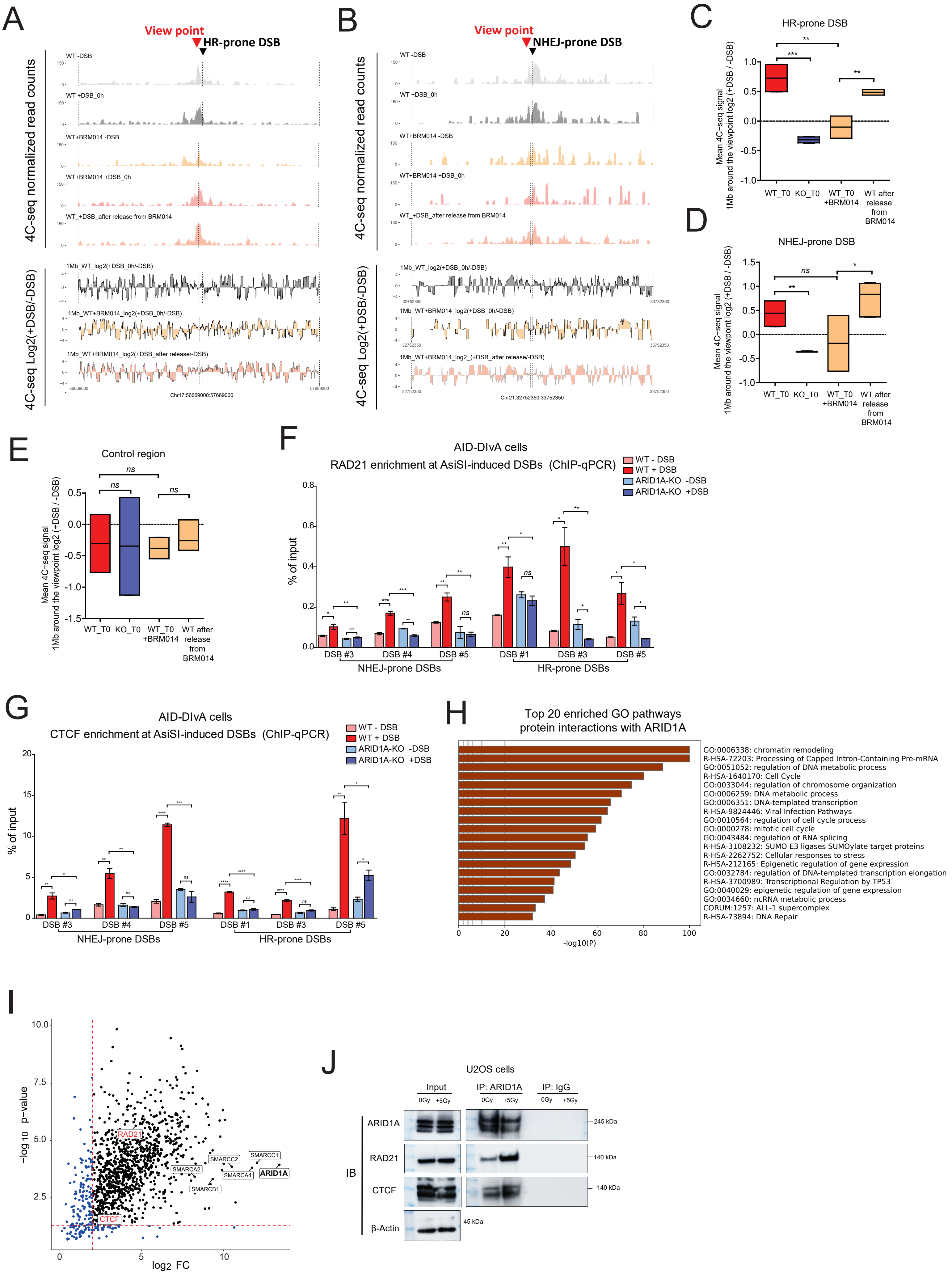

A
View point
HR-prone DSB
4C-seq normalized read counts
WT -DSB
WT +DSB_0h
WT+BRM014 -DSB
WT+BRM014 +DSB_0h
WT_+DSB_after release from BRM014
4C-seq Log2(+DSB/-DSB)
1Mb_WT_log2(+DSB_0h/-DSB)
1Mb_WT+BRM014_log2(+DSB_0h/-DSB)
1Mb_WT+BRM014_log2(+DSB_after release/-DSB)
Chr17:56669000:57669000
B
View point
NHEJ-prone DSB
4C-seq normalized read counts
WT -DSB
WT +DSB_0h
WT+BRM014 -DSB
WT+BRM014 +DSB_0h
WT_+DSB_after release from BRM014
4C-seq Log2(+DSB/-DSB)
1Mb_WT_log2(+DSB_0h/-DSB)
1Mb_WT+BRM014_log2(+DSB_0h/-DSB)
1Mb_WT+BRM014_log2_(+DSB_after release/-DSB)
Chr21:32752350:33752350
C
HR-prone DSB
Mean 4C−seq signal
1Mb around the viewpoint log2 (+DSB / -DSB)
WT_T0
KO_T0
WT_T0 +BRM014
WT after release from BRM014
D
NHEJ-prone DSB
Mean 4C−seq signal
1Mb around the viewpoint log2 (+DSB / -DSB)
WT_T0
KO_T0
WT_T0 +BRM014
WT after release from BRM014
E
Control region
Mean 4C−seq signal
1Mb around the viewpoint log2 (+DSB / -DSB)
WT_T0
KO_T0
WT_T0 +BRM014
WT after release from BRM014
F
AID-DIvA cells
RAD21 enrichment at AsiSI-induced DSBs (ChIP-qPCR)
WT - DSB
WT + DSB
ARID1A-KO -DSB
ARID1A-KO +DSB
% of input
DSB #3
DSB #4
DSB #5
DSB #1
DSB #3
DSB #5
NHEJ-prone DSBs
HR-prone DSBs
G
AID-DIvA cells
CTCF enrichment at AsiSI-induced DSBs (ChIP-qPCR)
WT - DSB
WT + DSB
ARID1A-KO -DSB
ARID1A-KO +DSB
% of input
DSB #3
DSB #4
DSB #5
DSB #1
DSB #3
DSB #5
NHEJ-prone DSBs
HR-prone DSBs
H
Top 20 enriched GO pathways
protein interactions with ARID1A
GO:0006338: chromatin remodeling
R-HSA-72203: Processing of Capped Intron-Containing Pre-mRNA
GO:0051052: regulation of DNA metabolic process
R-HSA-1640170: Cell Cycle
GO:0033044: regulation of chromosome organization
GO:0006259: DNA metabolic process
GO:0006351: DNA-templated transcription
R-HSA-9824446: Viral Infection Pathways
GO:0010564: regulation of cell cycle process
GO:0000278: mitotic cell cycle
GO:0043484: regulation of RNA splicing
R-HSA-3108232: SUMO E3 ligases SUMOylate target proteins
R-HSA-2262752: Cellular responses to stress
R-HSA-212165: Epigenetic regulation of gene expression
GO:0032784: regulation of DNA-templated transcription elongation
R-HSA-3700989: Transcriptional Regulation by TP53
GO:0040029: epigenetic regulation of gene expression
GO:0034660: ncRNA metabolic process
CORUM:1257: ALL-1 supercomplex
R-HSA-73894: DNA Repair
-log10(P)
I
−log 10 p−value
log2 FC
RAD21
CTCF
SMARCC2
SMARCC1
SMARCA2
SMARCA4
ARID1A
SMARCB1
J
U2OS cells
Input
IP: ARID1A
IP: IgG
0Gy
+5Gy
ARID1A
RAD21
CTCF
β-Actin
IB
245 kDa
140 kDa
140 kDa
45 kDa

Figure S3

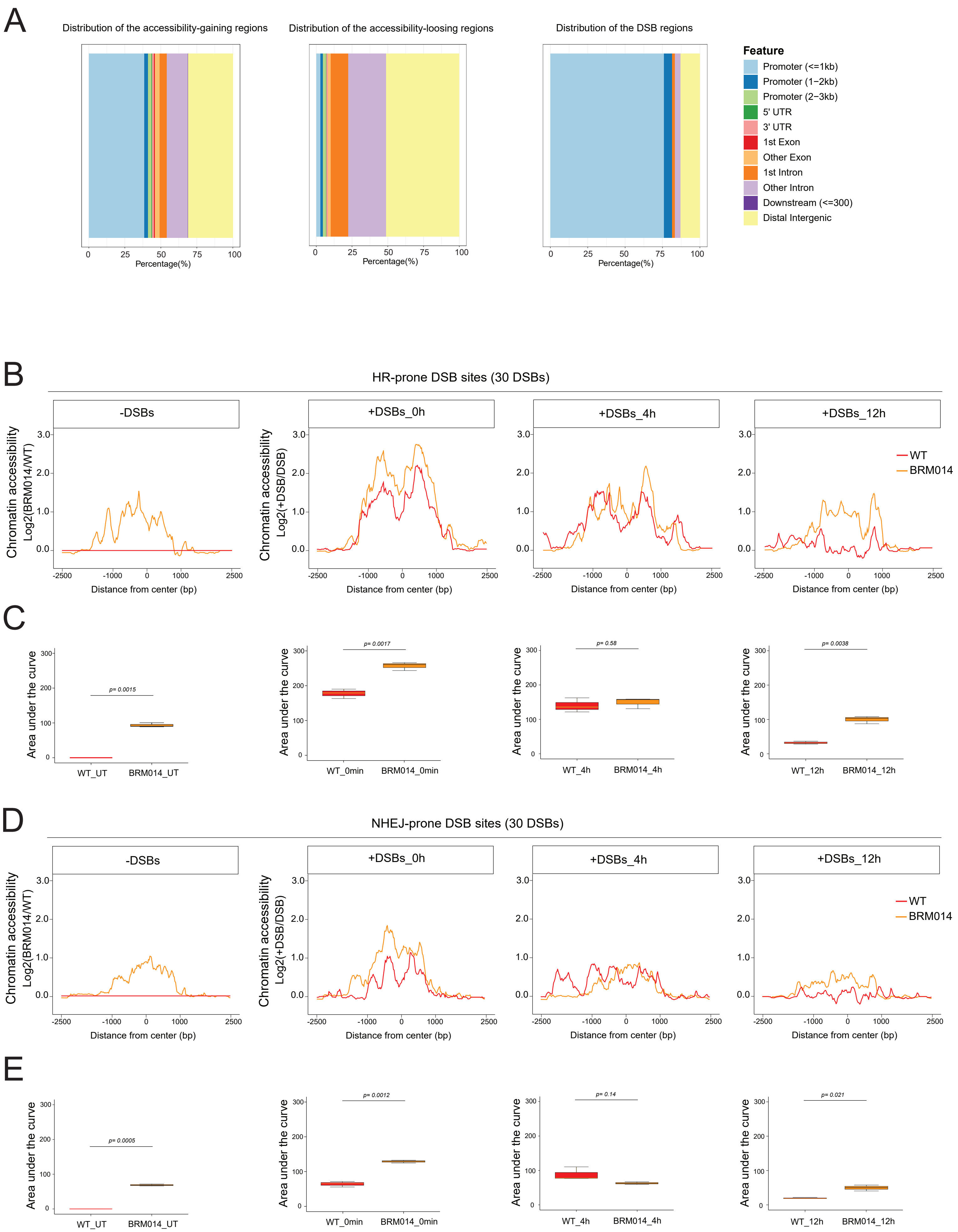
A
Distribution of the accessibility-gaining regions
Distribution of the accessibility-loosing regions
Distribution of the DSB regions
Percentage(%)
Feature
Promoter (<=1kb)
Promoter (1−2kb)
Promoter (2−3kb)
5' UTR
3' UTR
1st Exon
Other Exon
1st Intron
Other Intron
Downstream (<=300)
Distal Intergenic
B
HR-prone DSB sites (30 DSBs)
-DSBs
+DSBs_0h
+DSBs_4h
+DSBs_12h
Chromatin accessibility Log2(BRM014/WT)
Chromatin accessibility Log2(+DSB/DSB)
Distance from center (bp)
WT
BRM014
C
Area under the curve
p= 0.0015
p= 0.0017
p= 0.58
p= 0.0038
WT_UT
BRM014_UT
WT_0min
BRM014_0min
WT_4h
BRM014_4h
WT_12h
BRM014_12h
D
NHEJ-prone DSB sites (30 DSBs)
-DSBs
+DSBs_0h
+DSBs_4h
+DSBs_12h
E
p= 0.0005
p= 0.0012
p= 0.14
p= 0.021

Figure S4

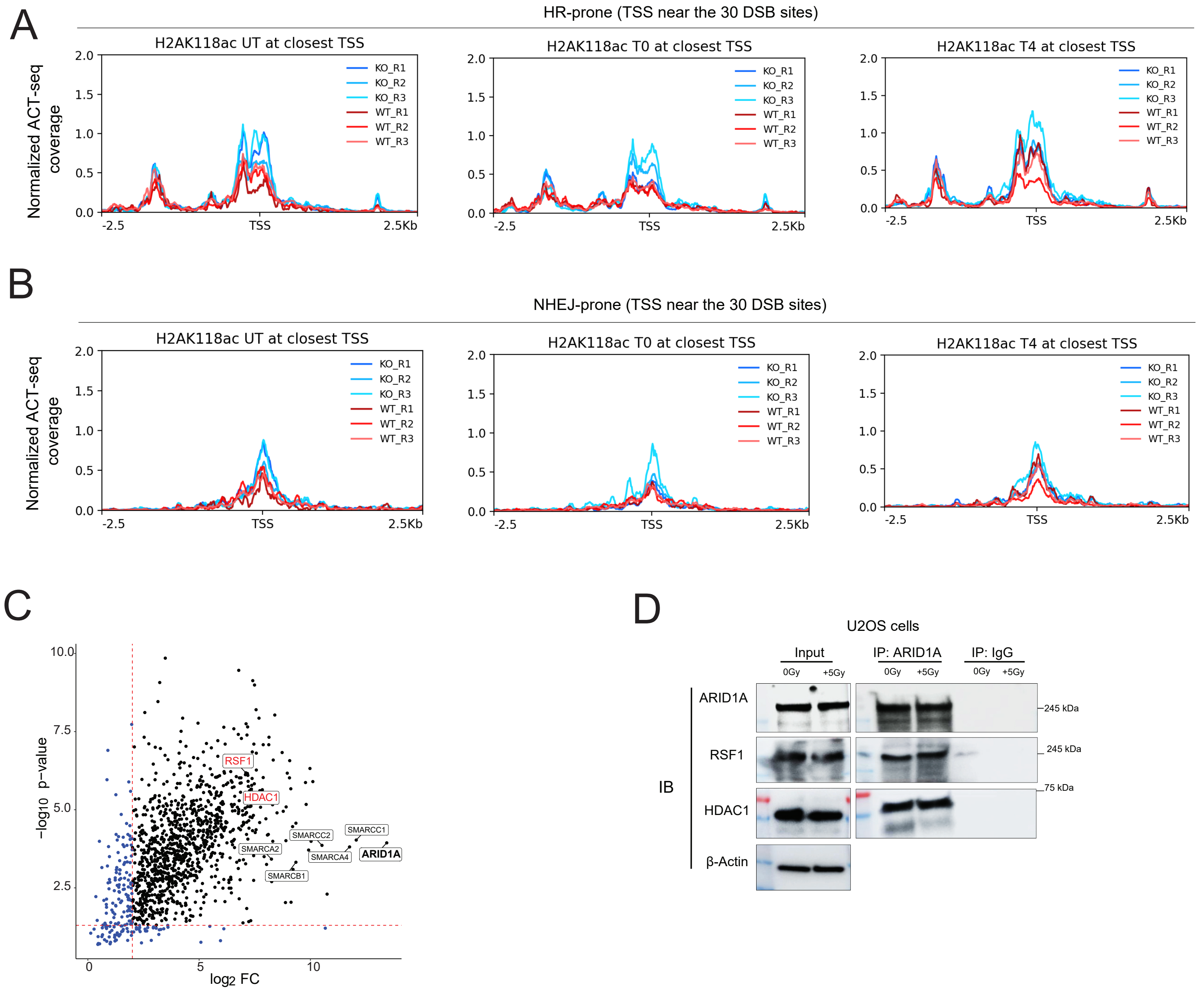
A
HR-prone (TSS near the 30 DSB sites)
H2AK118ac UT at closest TSS
H2AK118ac T0 at closest TSS
H2AK118ac T4 at closest TSS
Normalized ACT-seq coverage
KO_R1
KO_R2
KO_R3
WT_R1
WT_R2
WT_R3
-2.5
TSS
2.5Kb
B
NHEJ-prone (TSS near the 30 DSB sites)
H2AK118ac UT at closest TSS
H2AK118ac T0 at closest TSS
H2AK118ac T4 at closest TSS
Normalized ACT-seq coverage
C
−log10 p−value
log2 FC
RSF1
HDAC1
SMARCC2
SMARCC1
SMARCA2
SMARCA4
ARID1A
SMARCB1
D
U2OS cells
Input
IP: ARID1A
IP: IgG
0Gy
+5Gy
IB
ARID1A
RSF1
HDAC1
β-Actin
245 kDa
75 kDa

Figure S5

A

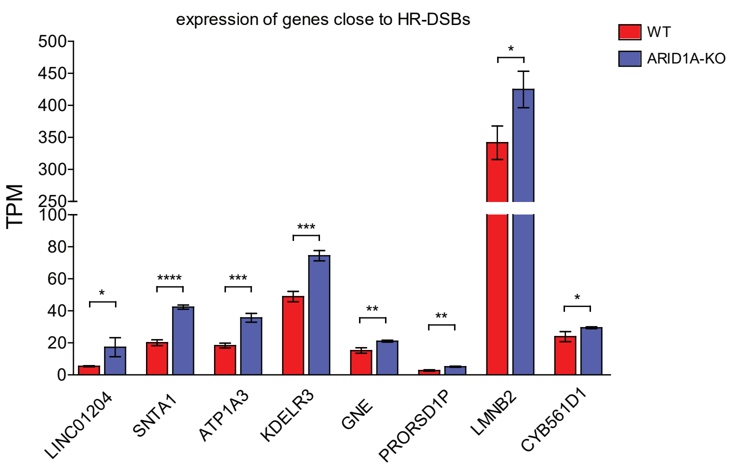
expression of genes close to HR-DSBs
WT
ARID1A-KO
TPM
500
450
400
350
300
250
100
80
60
40
20
0
*
****
***
***
**
**
*
*
LINC01204
SNTA1
ATP1A3
KDELR3
GNE
PRORSD1P
LMNB2
CYB561D1

B

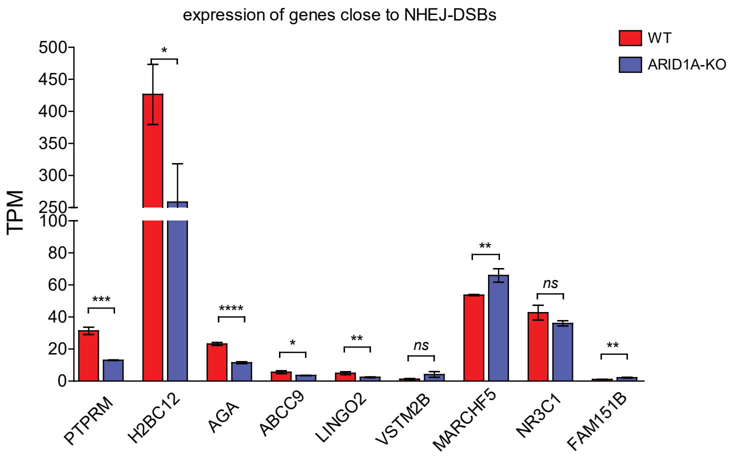
expression of genes close to NHEJ-DSBs
WT
ARID1A-KO
TPM
500
450
400
350
300
250
100
80
60
40
20
0
***
*
****
*
**
ns
**
ns
**
PTPRM
H2BC12
AGA
ABCC9
LINGO2
VSTM2B
MARCHF5
NR3C1
FAM151B

Figure S6

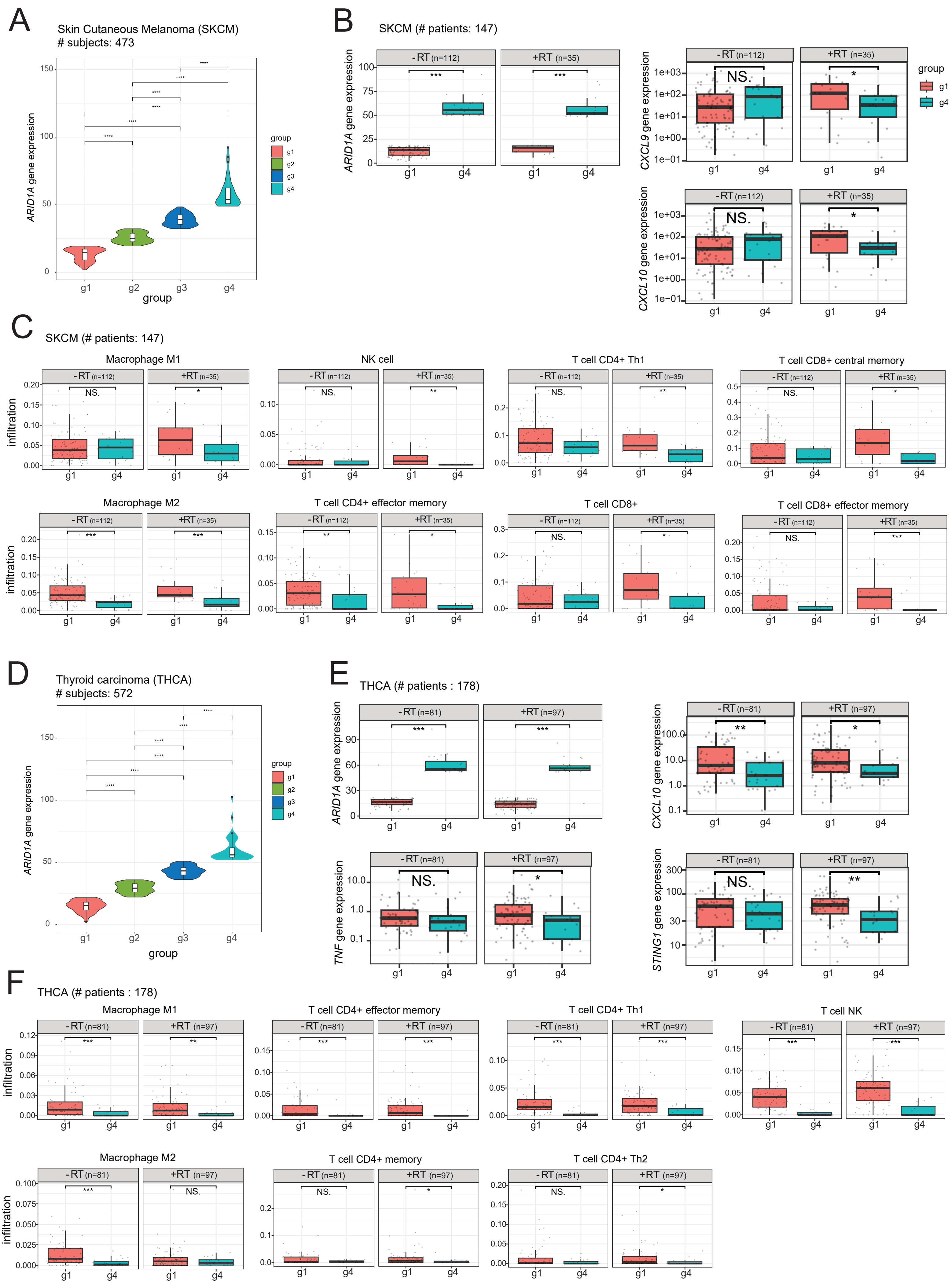
A
Skin Cutaneous Melanoma (SKCM)
subjects: 473
ARID1A gene expression
group
g1
g2
g3
g4
B
SKCM (# patients: 147)
ARID1A gene expression
CXCL9 gene expression
CXCL10 gene expression
-RT (n=112)
+RT (n=35)
NS.
C
SKCM (# patients: 147)
Macrophage M1
NK cell
T cell CD4+ Th1
T cell CD8+ central memory
Macrophage M2
T cell CD4+ effector memory
T cell CD8+
T cell CD8+ effector memory
infiltration
D
Thyroid carcinoma (THCA)
subjects: 572
E
THCA (# patients : 178)
-RT (n=81)
+RT (n=97)
CXCL10 gene expression
TNF gene expression
STING1 gene expression
F
THCA (# patients : 178)
Macrophage M1
T cell CD4+ effector memory
T cell CD4+ Th1
T cell NK
Macrophage M2
T cell CD4+ memory
T cell CD4+ Th2

Figure S7

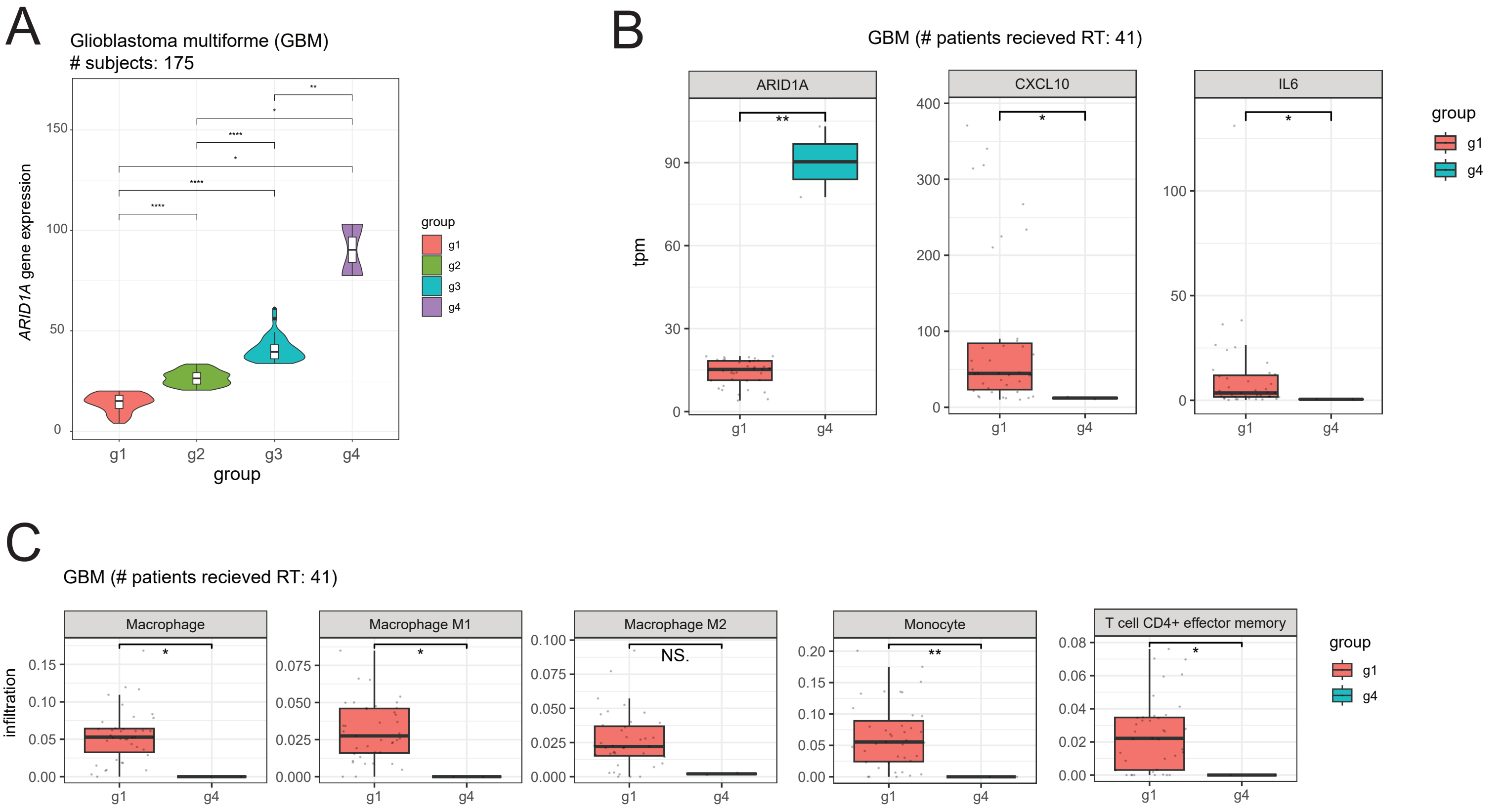
A
Glioblastoma multiforme (GBM)
subjects: 175
ARID1A gene expression
group
g1
g2
g3
g4
B
GBM (# patients recieved RT: 41)
ARID1A
CXCL10
IL6
tpm
NS.
C
GBM (# patients recieved RT: 41)
Macrophage
Macrophage M1
Macrophage M2
Monocyte
T cell CD4+ effector memory
infiltration
