# Supplementary material for: ARID1A regulates DNA repair through chromatin organization and its deficiency triggers DNA damage-mediated anti-tumor immune response
Source: Nucleic Acids Res. 2024 Apr 8;52(10):5698–719. doi: 10.1093/nar/gkae233 (PMC11162808; doi:10.1093/nar/gkae233)
Supplement: gkae233_Supplemental_Files [file gkae233_supplemental_files.zip › Supplementary Table S1 (Oligonucleotides).pdf]

Supplementary Table S1: Sequences of oligonucleotides

Sequences of individual small interfering RNAs

PRODUCT	individual siRNAs	Sequence
siGENOME SET OF 4 UPGRADE	siARID1A#1	GCAACGACAUGAUUCCUAU
	siARID1A#2	GAAUAGGGCCUGAGGGAAA
	siARID1A#3	AGAUGUGGGUGGACCGUUA
	siARID1A#4	UAGUAUGGCUGGCAUGAUC.
siGENOME SET OF 4 UPGRADE	Non-targeting#1	UGGUUUACAUGUCGACUAA
	Non-targeting#2	UGGUUUACAUGUUGUGUGA
	Non-targeting#3	UGGUUUACAUGUUUUCUGA
	Non-targeting#4	UGGUUUACAUGUUUUCCUA

Sequences of individual primers

Purpose	Target region	Orientation	Sequence
RT-qPCR	GAPDH	forward	AGCCACATCGCTCAGACAC
		reverse	GCCCAATACGACCAAATCC
	INF-A	forward	TGATGCTCCTGGCACAAATG
		reverse	GGATCAGCTCATGGAGGACA
	IL-6	forward	GATGAGTACAAAAGTCCTGATCCA
		reverse	CTGCAGCCACTGGTTCTGT
	CXCL9	forward	CTGTTCCTGCATCAGCACCAAC
		reverse	TGAACTCCATTCTTCAGTGTAGCA
	CXCL10	forward	GGTGAGAAGAGATGTCTGAATCC
		reverse	GTCCATCCTTGGAAGCACTGCA
ChIP-qPCR	NHEJ-DSB-1	forward	TCCCCTGTTTCTCAGCACTT
		reverse	CTTCTGCTGTTCTGCGTCCT
	NHEJ-DSB-2	forward	ATCGGGCCAATCTCAGAGG
		reverse	GCGACGCTAACGTTAAAGCA
	NHEJ-DSB-3	forward	GGAAGGAGGGGCTACTAGGG
		reverse	GAAAGCCCCATTCAGTTTGA
	NHEJ-DSB-4	forward	CGTGGGGAAGTTAAGGAACA
		reverse	CCCCATCACGTTAACCAAAC
	NHEJ-DSB-5	forward	GGGGAAATTGGGCATTAGTT
		reverse	TTCGCCTCTTGGTTCTCTGT
	HR-DSB-1	forward	TATGGGACCAAGCGAGTAGG
		reverse	GCCTCACACACACACCCATA
	HR-DSB-2	forward	GGGACAGCGCGTACTTTG
		reverse	TCGCTAGGCCCAGCAGTT
	HR-DSB-3	forward	GTCAGTATGGCCCCAGAGTC
		reverse	ACGGCTGATGGACTTAGACG
	HR-DSB-4	forward	GAGGAACCATTCGGACAAGA
		reverse	CTGACCAAGGAAGCCTCAAG
	HR-DSB-5	forward	GGGTATGGAGCTGCCTCTAA
		reverse	GACAAAGATGGCTGGAGGAG
	HR-DSB-at the 4C viewpoint	forward	CCGCCAGAAAGTTTCCTAGA
		reverse	CTCACCCTTGCAGCACTTG
	NHEJ-DSB-at the 4C viewpoint	forward	CCTAGCTGAGGTCGGTGCTA
		reverse	GAAGAGTGAGGAGGGGGAGT
	CTRL-noDSB-at the 4C viewpoint	forward	AGCACATGGGATTTTGCAGG
		reverse	TTCCCTCCTTTGTGTCACCA

Sequences of sBLISS primers

Purpose	adaptor	Orientation	Sequence
sBLISS adaptors	AsiSI_BLISS_#1	forward	/5Phos/CGCCTCACACGNNNNNNNNGATCGTCGGACTGTAGAACTCTGAACCCCTATAGTGAGTCGTATTACCGGCCTCAATCGAA
		reverse	CGATTGAGGCCGGTAATACGACTCACTATAGGGGTTCAGAGTTCTACAGTCCGACGATCNNNNNNNNCGTGTGAGGCGAT
	AsiSI_BLISS_#4	forward	/5Phos/CGCTGAGTTAGNNNNNNNNGATCGTCGGACTGTAGAACTCTGAACCCCTATAGTGAGTCGTATTACCGGCCTCAATCGAA
		reverse	CGATTGAGGCCGGTAATACGACTCACTATAGGGGTTCAGAGTTCTACAGTCCGACGATCNNNNNNNNCTAACTCAGCGAT
	AsiSI_BLISS_#10	forward	/5Phos/CGCTCGGATTCNNNNNNNNGATCGTCGGACTGTAGAACTCTGAACCCCTATAGTGAGTCGTATTACCGGCCTCAATCGAA
		reverse	CGATTGAGGCCGGTAATACGACTCACTATAGGGGTTCAGAGTTCTACAGTCCGACGATCNNNNNNNNGAATCCGAGCGAT
	AsiSI_BLISS_#13	forward	/5Phos/CGCCGAGATTCNNNNNNNNGATCGTCGGACTGTAGAACTCTGAACCCCTATAGTGAGTCGTATTACCGGCCTCAATCGAA
		reverse	CGATTGAGGCCGGTAATACGACTCACTATAGGGGTTCAGAGTTCTACAGTCCGACGATCNNNNNNNNGAATCTCGGCGAT
	AsiSI_BLISS_#15	forward	/5Phos/CGCAATGATTCNNNNNNNNGATCGTCGGACTGTAGAACTCTGAACCCCTATAGTGAGTCGTATTACCGGCCTCAATCGAA
		reverse	CGATTGAGGCCGGTAATACGACTCACTATAGGGGTTCAGAGTTCTACAGTCCGACGATCNNNNNNNNGAATCATTGCGAT
	AsiSI_BLISS_#17	forward	/5Phos/CGCAGTATCCGNNNNNNNNGATCGTCGGACTGTAGAACTCTGAACCCCTATAGTGAGTCGTATTACCGGCCTCAATCGAA
		reverse	CGATTGAGGCCGGTAATACGACTCACTATAGGGGTTCAGAGTTCTACAGTCCGACGATCNNNNNNNNCGGATACTGCGAT
sBLISS primers	RTP	NA	GCCTTGGCACCCGAGAATTCCA
	RP1 common primer	NA	AATGATACGGCGACCACCGAGATCTACACGTTCAGAGTTCTACAGTCCGA
	RA3	NA	/5rApp/TGGAATTCTCGGGTGCCAAGG/3SpC3/
	RPIX Index 6	NA	CAAGCAGAAGACGGCATACGAGATATTGGCGTGACTGGAGTTCCTTGGCACCCGAGAATTCCA
	RPIX Index 12	NA	CAAGCAGAAGACGGCATACGAGATTACAAGGTGACTGGAGTTCCTTGGCACCCGAGAATTCCA

Sequences of 4C viewpoint primers

Purpose	adaptor	Orientation	Sequence
4C-seq	DSB2_4C_NlaIII_F	NA	GTCTCGTGGGCTCGGAGATGTGTATAAGAGACAGAAGCTAATTCTGAGTTACATACATTATAGACATG
	DSB2_4C_DpnII_R	NA	TCGTCGGCAGCGTCAGATGTGTATAAGAGACAGTCCTTACGATTATTTGTGAATTTTGAAAATAG
	cont_4C_NlaIII_F	NA	GTCTCGTGGGCTCGGAGATGTGTATAAGAGACAGCACCTTCGCTGTACCTTTGCCACATG
	cont_4C_DpnII_R	NA	TCGTCGGCAGCGTCAGATGTGTATAAGAGACAGTCCTCAGGTTATCATCCCAATGGTAAC
	DSB3_4C_DpnII_F	NA	GTCTCGTGGGCTCGGAGATGTGTATAAGAGACAGGATTACGTAGAAGGGTGCCCTGAGTAGATC
	DSB3_4C_NlaIII_R	NA	TCGTCGGCAGCGTCAGATGTGTATAAGAGACAGAAGGCAAATGATAACCCTGTGCCCAAGCCT

Sequences of primers used in translocation assay

Purpose	adaptor	Orientation	Sequence
translocation assay (qPCR)	MIS12	forward	GACTGGCATAAGCGTCTTCG
	TRIM37	reverse	TCTGAAGTCTGCGCTTTCCA
	LINC00217	forward	GGAAGCCGCCCAGAATAAGA
	LYRM2	reverse	TCTGAAGTCTGCGCTTTCCA
	TRIM37	forward	AATTCGCAAACACCAACCGT
	RBMXL1	reverse	GCCAATGGAGTTCCCTGAGTC
	Control_chr1	forward	AGCACATGGGATTTTGCAGG
	Control_chr1	reverse	TTCCCTCCTTTGTGTCACCA
	Control_chr17	forward	ACAGTGGGAGACAGAAGAGC
	Control_chr17	reverse	CTCCATCATCGCACCCTTTG
