# Supplementary material for: ARID1A regulates DNA repair through chromatin organization and its deficiency triggers DNA damage-mediated anti-tumor immune response
Source: Nucleic Acids Res. 2024 Apr 8;52(10):5698–719. doi: 10.1093/nar/gkae233 (PMC11162808; doi:10.1093/nar/gkae233)
Supplement: gkae233_Supplemental_Files [file gkae233_supplemental_files.zip › Supplementary Table S2_annotation_DSBs.pdf]

Supplementary Table S2: Annotation of all DSBs

DSB genomic ID	chromosome	start	end	closest gene	location to promoter
HR_geID_1	chr1	9649446	9649452	TMEM201	downstream
HR_geID_2	chr1	89458597	89458603	KYAT3	within promoter
HR_geID_3	chr1	110036700	110036706	CYB561D1	within promoter
HR_geID_4	chr2	55509101	55509107	PRORSD1P	upstream
HR_geID_5	chr2	85822594	85822600	RNF181	upstream
HR_geID_6	chr3	52232163	52232169	ALAS1	within promoter
HR_geID_7	chr3	98618164	98618171	NA	NA
HR_geID_8	chr3	99536965	99536971	HP09053	within promoter
HR_geID_9	chr9	36258514	36258520	GNE	downstream/intragenic
HR_geID_10	chr9	127532105	127532112	NA	NA
HR_geID_11	chr9	130693171	130693177	PIP5KL1	upstream
HR_geID_12	chr9	130889407	130889414	NA	NA
HR_geID_13	chr12	13154718	13154724	GPRC5D-AS1	within promoter
HR_geID_14	chr12	121975058	121975064	KDM2B	downstream/intragenic
HR_geID_15	chr14	54955826	54955832	GMFB	upstream
HR_geID_16	chr17	5390221	5390227	MIS12	downstream
HR_geID_17	chr17	38137473	38137479	PSMD3	downstream/intragenic
HR_geID_18	chr17	57184297	57184303	TRIM37	upstream
HR_geID_19	chr17	80250840	80250847	NA	NA
HR_geID_20	chr19	2456094	2456100	LMNB2	downstream
HR_geID_21	chr19	41903743	41903749	BCKDHA	within promoter
HR_geID_22	chr19	42497856	42497862	ATP1A3	downstream
HR_geID_23	chr20	1207616	1207622	RAD21L1	downstream
HR_geID_24	chr20	30946313	30946319	ASXL1	within promoter
HR_geID_25	chr20	32032087	32032093	SNTA1	upstream
HR_geID_26	chr20	42087118	42087124	SRSF6	downstream/intragenic
HR_geID_27	chr21	46221790	46221796	UBE2G2	upstream
HR_geID_28	chr22	20850308	20850314	KLHL22	upstream
HR_geID_29	chr22	38864102	38864108	KDELR3	within promoter
HR_geID_30	chrX	45366394	45366400	LINC01204	downstream/intragenic

DSB genomic ID	chromosome	start	end	closest gene	location to promoter
NHEJ_geID_1	chr2	43358339	43358345	LINC02580	upstream far away/intergenic
NHEJ_geID_2	chr2	68384749	68384755	WDR92/PNO1	Upstream/intergenic
NHEJ_geID_3	chr2	74734762	74734768	PCGF1	downstream/intragenic
NHEJ_geID_4	chr2	208030728	208030734	MIR7845	downstream/intragenic
NHEJ_geID_5	chr4	178363576	178363582	AGA	within promoter
NHEJ_geID_6	chr5	79784140	79784146	FAM151B	downstream/intragenic
NHEJ_geID_7	chr5	142785050	142785056	NR3C1	downstream/intragenic
NHEJ_geID_8	chr6	27145367	27145373	H2BC12	upstream far away/intergenic
NHEJ_geID_9	chr6	31105427	31105434	NA	NA
NHEJ_geID_10	chr6	49917583	49917589	DEFB114	downstream far away/intergenic
NHEJ_geID_11	chr6	67704021	67704027	NA	downstream far away/intergenic
NHEJ_geID_12	chr6	90348187	90348193	LYRM2	downstream/intragenic
NHEJ_geID_13	chr6	135819348	135819354	LINC00271	downstream/intragenic
NHEJ_geID_14	chr6	149888106	149888112	GINM1	downstream/intragenic
NHEJ_geID_15	chr7	75807507	75807513	SRRM3	upstream far away/intergenic
NHEJ_geID_16	chr7	92861491	92861497	HEPACAM2	within promoter
NHEJ_geID_17	chr8	66546348	66546354	ARMC1	downstream/intragenic
NHEJ_geID_18	chr9	29212800	29212806	LINGO2	downstream/intragenic
NHEJ_geID_19	chr10	94051015	94051021	MARCHF5	downstream/intragenic
NHEJ_geID_20	chr11	24518476	24518482	LUZP2	Upstream/intergenic
NHEJ_geID_21	chr11	75525761	75525767	UVRAG	Upstream/intergenic
NHEJ_geID_22	chr12	22093988	22093995	NA	NA
NHEJ_geID_23	chr12	130091881	130091887	ABCC9	upstream far away/intergenic
NHEJ_geID_24	chr13	105238552	105238558	NA	NA
NHEJ_geID_25	chr18	7566713	7566719	PTPRM	Upstream/intergenic
NHEJ_geID_26	chr19	30019488	30019494	VSTM2B	downstream/intragenic
NHEJ_geID_27	chr20	20032925	20032931	CRNKL1	downstream/intragenic
NHEJ_geID_28	chr21	33245519	33245525	HUNK	within promoter
NHEJ_geID_29	chrX	1510671	1510678	NA	NA
NHEJ_geID_30	chrX	72783103	72783109	CHIC1	within promoter
