# Supplementary material for: ARID1A regulates DNA repair through chromatin organization and its deficiency triggers DNA damage-mediated anti-tumor immune response
Source: Nucleic Acids Res. 2024 Apr 8;52(10):5698–719. doi: 10.1093/nar/gkae233 (PMC11162808; doi:10.1093/nar/gkae233)
Supplement: gkae233_Supplemental_Files [file gkae233_supplemental_files.zip › Supplementary Table S3_Identified interaction_Volcano plot.pdf]

Supplementary Table S3: list of all detected interactions

Gene names	log2FoldChange	pvalue (-log10)
ARID1A	13.43	3.94
SMARCC1	12.06	4.03
SMARCA4	11.76	3.81
PYCR1;PIG45	10.75	2.3
HIST1H2BI;HIST1H2BK;HIST1H2BN;HIST1H2BD;HIST1H2BM;HIST1H2BL;HIST1H2BH;HIST2H2BF;HIST1H2BC;H2BFS;HIST1H2BA	10.67	1.21
SMARCC2	10.52	3.86
SMARCE1	10.43	3.42
DPF2	10.22	3.49
MKI67	10.11	5.9
SMARCD2	10.05	3.53
PYCR2	10.03	3.99
SPECC1	9.98	6.32
SMARCD1	9.92	3.71
AMOT	9.81	5.17
RBM14	9.78	5.87
KHDRBS1	9.35	4.57
SMARCB1	9.35	3.32
H3F3B;H3F3A;HIST3H3	9.22	3.09
ACTL6A;BAF53A	9.21	4.16
SRRM2;KIAA0324	9.2	4.46
CHD4	9.13	2.04
SFPQ	9.12	5.67
CSE1L	9.07	6.57
ADAR	9.06	6.02
NONO	8.96	6.17
HNRNPC;hCG_1641229	8.91	5.09
IMPDH2	8.9	4.01
SMARCA5	8.88	2.03
YLPM1;FLJ00353	8.78	4.95
RBBP4	8.73	6.83
HCTP4;TPX2	8.5	4.9
DDX3X;DDX3Y	8.4	8.03
ELAVL1	8.33	6.59
CENPF	8.31	3.99
SLC25A5	8.3	5.16
TRIM28	8.26	2.7
SMARCA2	8.25	3.42
PRPF6	8.14	7.32
SSRP1	8.13	7.08
ZMYM4	8.11	4.21
SAFB	8.07	4.19
BCS1L	8.03	3.25

MDC1	8	4.71
LBR	7.92	3.5
CLTC	7.88	6.03
U2SURP	7.88	7.08
ACIN1;DKFZp667N107	7.84	5.64
NOLC1	7.84	5.58
NPM1	7.76	5.08
GTF3C1;DKFZp686A111;DKFZp686O0870	7.73	5.61
HEL-S-91;SRSF6	7.72	4.59
TOP2A	7.69	7.93
ADNP	7.61	5.47
MTA2	7.57	6.81
SPEN	7.57	6.27
DHX9	7.56	4.36
HIST2H3PS2	7.56	8.2
LMNB1	7.52	4.27
MSH2	7.49	8.99
POGZ	7.48	5.83
CEP170	7.46	7.1
SMARCD3	7.45	2.74
SPTAN1;DKFZp564P0562	7.45	3.53
NKRF;NRF	7.44	4.82
EWSR1	7.41	9.13
KMT2A	7.4	4.33
TP53BP1	7.4	5.39
XRN2	7.39	6.85
CBX3	7.38	6
HIST1H2BJ;HIST2H2BE;HIST1H2BB;HIST1H2BO;HIST3H2BB	7.38	4.75
IRF2BP1	7.36	5.78
CPSF1	7.35	5.66
SPTBN1	7.33	5.12
HEL-S-69p;PPIA	7.32	5.11
SMC1A;DKFZp686L19178	7.32	1.45
SMC2L1;SMC2	7.29	4.77
TMPO	7.28	5.1
WDR57;SNRNP40	7.27	5.61
DOCK7	7.24	4.41
CHERP	7.23	4.65
SIN3A	7.23	1.42
HDAC1	7.21	5.75
BAZ1B	7.2	5.91
RUVBL1	7.2	3.68
CYFIP1	7.19	4.06
SMCHD1	7.19	6.1
GATAD2A	7.18	7.65
GATAD2B	7.18	5.1
PRPF40A	7.17	6.16

RAD50	7.15	7.39
NAT10	7.14	1.84
TAF15	7.14	4.6
KIF22	7.13	4.9
PRPF19	7.13	4.21
RIF1	7.13	4.91
TCF20	7.11	7.75
RSF1	7.1	6.18
SMARCB1	7.09	3.27
H2AFY	7.08	6.12
TDP43;TARDBP	7.08	5.26
MCM7	7.07	5.05
RUVBL2	7.07	5.05
WIZ	7.07	4.85
GTF2I	7.01	4.57
CHAMP1	6.99	4.53
HNRNPA0	6.99	4.6
TPR	6.99	3.4
U2AF1;U2AF1L4	6.97	1.38
RBMX	6.96	3.83
ZNF638	6.96	4.83
TCOF1	6.95	3.61
TRRAP	6.95	4.97
RBBP7	6.92	4.48
TBL3	6.9	7.28
NIPBL	6.89	5.86
S100A8	6.89	3.63
CSNK2A1;CSNK2A3	6.88	6.02
ALDH18A1	6.86	4.36
PTBP1	6.86	5.56
GTF3C3	6.84	5.85
PLEC;PLEC1	6.84	2.76
ANLN	6.81	3.38
BAZ1A	6.81	6.02
SF3B3	6.8	5.29
SS18L1	6.8	2.15
FASN	6.78	9.46
HNRNPR;HNRPR	6.78	4.94
RNF2	6.78	4.41
TUBB2C;TUBB4B	6.78	5.01
FIP1L1	6.76	4.88
RBM15	6.76	7.41
SRSF7	6.76	3.12
NUP155	6.72	5.08
STOML2	6.69	3.32
BPTF	6.67	6.06
SMC4	6.61	4.97
CHD1L	6.6	5.43
SRCAP	6.59	3.79
ILF3	6.58	4.05

MCM4	6.58	5.17
RBM17	6.57	5.73
HNRNPUL2;HNRNPUL2-BSCL2	6.56	7.59
SFRS14;SUGP2	6.54	4.37
DIDO1	6.53	4
RPRD2	6.53	4.01
PNN	6.51	4.34
ATAD3A	6.5	3.88
GTF3C5	6.5	4.96
HELLS	6.49	5.1
SFRS3;SRSF3	6.49	5.71
CPSF2	6.48	5.88
EP400	6.46	3.98
CDC5L	6.45	5.17
DHX15	6.45	2.34
SF3A1	6.44	5.93
HNRPA2B1;HNRNPA2B1	6.43	2.77
NCL	6.43	2.49
DPF1	6.42	2.84
WDR33	6.42	6.22
POLR2A	6.4	4.29
SRSF1	6.39	3.46
SS18;SYT-SSX2;SYT-SSX1;SYT	6.35	2.49
BCL7A	6.34	2.75
KMT2D	6.34	2.67
DBN1	6.29	5.86
PHF3	6.28	6.44
CDYL	6.25	3.94
GTF3C2	6.25	4.59
UBTF	6.22	5.81
IRS4	6.21	4.58
DKFZp686P17171;SERBP1	6.2	4.62
RCC1	6.19	4.44
DNMT1	6.18	6.72
CHD8	6.17	7.1
PRC1	6.16	4.44
SMARCC2	6.16	2.06
KIF4A	6.14	6.17
ZNF326	6.14	4.39
CHD7	6.12	4.33
HEL-S-270;ANXA2;ANXA2P2	6.12	6.22
RBM26	6.12	4.4
EEF1G	6.11	1.19
HIST1H1C;HIST1H1E;HIST1H1D	6.11	2.77
RNF40	6.11	7.64
PBRM1	6.09	5.77
ZFR	6.09	5.19
KIF23	6.08	4.38

NDUFA5	6.07	2.49
PRIC295;GCN1L1	6.06	3.58
ANAPC7	6.05	5.17
DDX1	6.05	5.26
HEL-S-133P;LDHA	6.05	4.14
MCM6	6.05	4.89
EHMT1	6.04	4.93
MAP1B;DKFZp686F1345	6.03	3.49
MTHFD1	6.03	4.42
CCDC137	6.02	4.12
CPNE3	6	4.53
HNRPD;HNRNPD	6	5.55
TTF2	5.99	4.2
BUB3	5.98	5.21
POLR2B	5.98	4.75
TUFM	5.98	4.46
BAP18;C17orf49;RNASEK-C17orf49	5.97	4.64
RFC3	5.97	5.48
API5	5.96	0.81
MED1	5.95	3.6
FUS	5.94	4.96
ZNF198;ZMYM2	5.92	6.09
CSTF1	5.91	5.07
HEL-S-72p;HSPA8	5.9	4.99
MRE11A	5.9	5.02
MBD3	5.89	5.44
ANAPC1	5.88	3.86
UQCRC2	5.88	2.67
ERH	5.87	4.76
ZNF687	5.87	5.07
PHF14	5.86	4.77
HNRPF;HNRNPF	5.85	4.57
SART1	5.85	4.39
INTS1;DKFZP586J0619	5.84	7.21
MCM5	5.84	6.71
SNRPD1	5.84	6.06
WDR5	5.83	4.64
H2AFV;H2AFZ	5.82	3.38
NOP58	5.82	4.33
BRD2	5.81	3.09
NUMA1	5.81	4.85
RTCB	5.81	4.72
CBX8	5.79	6.04
HEL25;CBX5	5.79	5.57
PPP1R10	5.79	4.34
PHB2	5.78	5.01
IRF2BP2	5.77	5.46
SF3B2;DKFZp781L0540	5.77	5.53
RSL1D1	5.76	3.23

DMAP1	5.75	4.47
NCKAP1	5.75	4.14
CPSF3	5.74	4.33
CSA2;IK	5.74	4.56
SNW1	5.74	6.04
ZC3H11A	5.74	4.8
NOC2L	5.73	4.81
PDCD11	5.73	2.08
WAC	5.73	2.87
TCP1	5.72	4.66
ARHGEF2	5.71	4.01
C1QBP	5.71	2.52
FAM208A	5.7	5.37
UBN2	5.7	3.61
CHAF1A	5.68	4.3
KIFC1	5.67	5.7
RPN1	5.67	4.43
CDC27	5.62	3.89
PRPF4B	5.61	3.63
CECR2	5.6	3.82
EHMT2	5.59	3.91
MAZ	5.59	3.83
SEPT7;Nbla02942;DKFZp686F17268	5.59	4.82
HMG20A	5.58	6.12
BCOR;BCOR-RARA	5.57	4.22
BAZ2A	5.56	3.59
NUP210	5.56	2.85
UACA	5.56	3.19
02-Sep	5.55	3.53
LRWD1	5.55	4.1
RFC5	5.55	6.98
ZMYM3	5.55	4.09
DDX20	5.54	4.88
SCML2	5.54	5.16
SRBD1	5.54	5.49
OGT	5.53	4.6
PRR12	5.53	4.28
DCTN1;DKFZp686I0746	5.51	3.48
HDAC2	5.51	4.12
RAI1	5.5	6.31
RFC2	5.5	4.48
SKIV2L2	5.5	5.55
SON	5.5	4.7
PHB;HEL-S-54e	5.49	4.32
ARID1B	5.48	3.82
CDC2;CDK1;DKFZp686L20222	5.48	1.27
RALY	5.48	4.43
THOC2	5.47	5.75

DDX48;EIF4A3	5.46	5.58
SMARCA1	5.46	5.74
RFC1	5.44	4.88
MED14	5.43	3.73
ZC3HAV1	5.43	5.25
SETD1A;hCG_1998636	5.41	3.75
MCM2	5.4	5.29
ZNF629	5.4	4.21
SPTBN2	5.39	4.3
UQCRC1	5.39	4.62
TUBG1;TUBG2	5.38	4.27
BRD4	5.37	4.12
DDX17	5.37	4.36
PLK1	5.36	5.8
CSNK2B;CSNK2B-LY6G5B-1181;CSNK2B-LY6G5B--991	5.35	3.98
CXXC1	5.35	3.36
HIRA	5.35	4.93
MDN1	5.35	4.55
RRP12	5.34	4.27
ZNF207;DKFZp761N202	5.34	4.92
SF1	5.33	7.12
RANBP2	5.32	4.67
CROCC;hCG_2007065;CROCCP3	5.31	2.29
SRSF10	5.31	5.79
SYNCRIP	5.31	3.89
PRPF8	5.3	5.1
TXLNA	5.3	4.77
RPL4	5.28	4.02
NUDT21	5.26	3.98
GTF3C4	5.25	3.56
LDHB	5.25	3.81
PSPC1	5.25	3.95
KIF2C	5.24	3.92
MAP4	5.24	3.88
CIZ1	5.23	3.49
RNF20	5.23	6.42
SNRPD2	5.23	4.63
DPF3	5.21	2.21
HK1	5.21	2.03
HNRNPLL;HNRPLL	5.21	3.94
SYMPK;SYMPK variant protein	5.21	3.44
NOC3L	5.2	4.35
RFC4	5.2	5.55
DCD	5.19	6.85
ORC2L;ORC2	5.19	4.3
PRPF38A	5.19	4.18
ZNF512;DKFZp666L156	5.19	4.02

ZNF644	5.19	3.68
COIL	5.17	5.91
TMEM113;WDR82	5.17	5.02
SAFB2	5.16	4.55
TUBB;XTP3TPATP1	5.15	2.64
SMC5	5.14	5.02
CHCHD3	5.13	4.1
NUP98	5.13	4.59
ANAPC5	5.11	5.01
NUP153	5.11	3.1
DIS3;KIAA1008	5.1	5.81
WTAP	5.1	6.06
C1orf57;NTPCR	5.09	3.85
KDM6A;UTX;DKFZp451J023	5.09	2.41
ABCF2	5.08	5.14
CAD	5.08	1.84
HEL-S-123m;ATP5A1	5.08	0.76
SLC25A3	5.08	4.66
ABI1	5.07	4.5
IMMT	5.06	1.63
SLTM	5.06	5.33
VRK1	5.05	4.33
ARID2	5.04	3.64
HIC2	5.04	5.96
MCM3;HCC5	5.04	5.73
COPA	5.03	3.77
HEL2;YWHAE;YWHAE/FAM22B fusion;YWHAE/FAM22A fusion	5.03	5.93
MORC2	5.03	3.15
VDAC2	5.02	3.02
SRSF9	5.01	4.71
GRWD1;DKFZp564C172	4.98	4.98
HEL-S-102;HSPB1	4.98	4.34
HNRPUL1;HNRNPUL1	4.98	5.1
MED12;TNRC11	4.98	3.31
MICAL3	4.98	4.29
CNN3	4.97	3.32
CPSF4	4.97	4.54
NCAPG2	4.97	4.73
ACTR1A	4.95	7.77
CPSF6	4.95	3.19
HACD3	4.95	4.62
RACGAP1	4.95	3.74
NUP205	4.94	4.16
RAN	4.94	6.64
DDX41	4.93	3.28
HEL-S-80p;PPP1CB	4.93	3.76
KDM1A	4.93	3.42
NCAPD3	4.93	4.7

SFXN1	4.93	3.79
MFAP1	4.92	5.77
MYL6	4.91	3.61
HCFC1	4.9	2.82
HNRNPDL;HNRPDL	4.9	5.72
TNKS1BP1	4.9	2.39
NUP93	4.89	5.3
INTS3	4.88	4.9
SUPT16H	4.88	2.87
HEL-S-124m;HSPA9	4.87	3.75
HEL-S-164nA;GANAB	4.87	3.66
HLTF	4.87	4.82
DKFZp686F18120;TRA2B	4.86	3.03
XPC	4.86	3.81
CYFIP2;DKFZp761H087	4.85	2.9
PCNA	4.84	3.76
YEATS2	4.83	2.04
CEP55	4.81	4.97
GLYR1;N-PAC	4.81	3.78
VAPB	4.81	4.06
FLJ10154;ARGLU1	4.8	2.73
MMTAG2	4.8	2.9
VPS72	4.8	4.1
ACTC1;ACTA2;ACTG2;ACTA1	4.79	4.08
EPRS	4.78	5.02
TOX4	4.78	4.11
ORC5L;ORC5	4.77	3.87
RNPS1	4.77	4.97
THRAP3	4.77	5.17
CRSP8;MED27	4.75	5.29
KAT7	4.75	4.36
TECR	4.75	3.11
ZC3H13	4.75	6.67
SFRS4;SRSF4	4.74	4.65
U5-116KD;EFTUD2;SNRP116	4.74	2.9
SCAF11;SFRS2IP	4.73	4.37
UTP18	4.73	3.93
AP2M1	4.72	4.98
MTA1	4.72	1.59
PUM1	4.72	4.25
DNCL1;DYNLL1	4.71	3.31
HDGFRP2	4.71	4.9
MSH3	4.71	4.26
WAPAL	4.69	4.88
ASCC3	4.68	4.06
EIF4A1;EIF4A2	4.68	5.22
MED17	4.68	3.21
hCG_1989366;NUP50	4.66	1.6
NBN	4.66	5.27
CPSF7	4.65	3.08

FAM208B	4.65	2.89
USP39	4.65	5.13
RAD21	4.64	5.64
CTBP1	4.63	4.48
FOXC1	4.62	6.15
HNRNPA3	4.62	1.61
MPHOSPH8	4.62	4.6
RING1	4.62	3.55
TXLNG	4.62	2.95
DDX52	4.61	5.04
CSNK2A2	4.6	4.19
DDX50;mcdrh;DKFZp761E0323	4.6	4.14
DKFZp686C21148;STAG2;DKFZp686P16143;DKFZp686I05169;DKFZp686P168	4.6	4.32
AHCTF1	4.59	3.9
SUPT6H	4.59	5.92
CBX2	4.58	6.4
PGAM5	4.58	2.88
RPN2	4.58	4.22
RPRD1B	4.58	3.42
S100A7	4.58	1.8
SMC6	4.58	3.9
SNRNP200	4.58	1.46
ORC3	4.57	3.6
NUP107	4.56	3.95
EIF3C;EIF3CL	4.54	3.2
INCENP	4.54	3.97
SNRPF	4.54	3.92
SNRPD3	4.52	3.36
11-Sep	4.51	3
EIF5A;EIF5AL1	4.51	5.8
THOC6	4.51	4.2
CAPRIN1	4.49	3.37
CKAP2	4.49	3.2
HNRNPL	4.49	2.65
CBX1	4.48	4.32
SUPT5H	4.48	6.09
BCLAF1	4.47	2.82
SF3A3	4.47	3.26
SF3B6	4.46	4.06
ZNF22	4.46	4.08
KIAA1429	4.45	5.02
SFRS5;SRSF5	4.45	4.53
ZNF24	4.45	3.59

HEL-S-61;KIF5B;KIF5B-RET(NM_020630)_K23;R12;KIF5B-RET(NM_020975)_K23;R12;KIF5B-RET(NM_020630)_K24;R11;KIF5B-ALK;KIF5B-RET(NM_020630)_K22;R12;KIF5B-RET(NM_020975)_K22;R12;KIF5B-ALK_K17;A20;KIF5B-RET(NM_020630)_K15;R12;KIF5B-RET(NM_020975)_K15;R12;KIF5B-RET(NM_020630)_K16;R12;KIF5B-RET(NM_020975)_K16;R12	4.44	2.18
SSR1	4.44	4.71
HEL-S-30;PKM;PKM2	4.43	3.73
RRP1B	4.42	1.43
TOP2B	4.42	1.52
BRD8	4.41	1.95
FLJ12949;KRI1	4.41	7.03
ZNF280C	4.41	3.9
MYEF2	4.4	4.11
QSER1	4.4	3.51
ZBTB10	4.4	3.07
BCAS2	4.39	5.18
DEK	4.39	3.61
UPF1	4.39	2.89
ZNF768;FLJ23436	4.39	2.01
ZZZ3	4.39	1.94
HAUS6	4.37	2.48
SURB7;MED21	4.37	2.88
ERCC2	4.36	2.52
STRAP	4.36	4.48
MGA	4.35	3.77
CTNND1;DKFZp781O2021	4.34	3.46
CWC22	4.33	6.5
FXR1	4.33	4.35
TBC1D10B	4.33	2.84
TBL1XR1	4.33	3.55
UBE2M	4.33	4.53
SRP14	4.32	4.84
EXOC4	4.31	5.41
PLRG1	4.31	3.37
BLM	4.3	3
PCF11	4.3	3.45
TRIOBP;DKFZp547M048	4.3	4.93

USP48	4.29	2.94
FOXK1;KIAA0415	4.28	2.37
INTS9	4.28	3.54
NSD1	4.28	4.15
POLDIP3	4.28	2.61
TOPBP1	4.28	3.35
PPP1CA	4.27	1.72
TFCP2	4.27	5.44
P15RS;RPRD1A	4.26	7.93
AHNAK	4.25	1.67
DACH1	4.25	5.15
LMNB2	4.24	3.67
MED8	4.24	3.41
MRGBP	4.24	3.83
DSC1	4.23	5.31
hCG_31253;FUBP3	4.22	2.77
BCL7C	4.21	1.92
CCNB1;CCNB1V	4.21	4.7
HOXD13	4.21	5.06
TADA3L;TADA3	4.21	3.76
MED20	4.2	4.41
MLLT4	4.2	2.91
DPY30;LOC84661	4.19	2.73
RPL6	4.19	4.36
RRP8	4.19	3.29
WDR43	4.19	7.51
DKC1	4.18	6.52
PAXIP1	4.18	3.39
AMOTL1	4.17	3.48
PPP2R1A	4.17	4.4
RBBP5	4.17	4.33
AHDC1	4.16	2.73
DYNC1H1	4.16	2.95
RBM15B	4.16	7.18
RBPJ;RBPSUH	4.16	3.91
SRI	4.15	4
ARID4B	4.14	4.82
EBF3;DKFZp667I0324;EBF1	4.14	5.84
IQGAP1;hCG_1991735	4.14	6.34
MYBL2	4.14	4.51
NES	4.14	2
DYNC2H1	4.13	4.16
EIF4G1;EIF4G1 variant protein	4.13	3.85
MED18	4.13	3.53
RPS8	4.13	3.5
C14orf166	4.11	4.69
MED28	4.11	6.46
DNAJA1;HDJ2	4.1	6.81
PRPS1;PRPS2	4.1	3.85

ZNF598	4.1	3.68
ERCC3	4.09	2.94
RBM4	4.09	3.4
AP2A1	4.08	4.15
HOXB9	4.08	3.12
PHF8	4.08	3.81
TUBGCP2	4.08	4.39
HEL103;SRP72	4.07	2.8
RPS13	4.07	5.21
TAF10	4.07	3.5
UBN1	4.07	2.99
GNL3	4.06	3.85
MYH9	4.06	2.81
LEMD3	4.05	5.15
H2AFY2	4.04	4.1
KIAA0020	4.04	3.58
PRPF4	4.04	3.85
ZNF609	4.04	2.39
CROP;LUC7L3	4.03	4.08
GNB2L1	4.03	3.51
HNRNPH2	4.03	5.84
RPL5	4.03	5.03
CABIN1	4.02	2.06
NVL	4.02	3.69
RB1	4.02	4.84
WDR75	4.02	4.59
CDC73	4.01	2
CDCA8	4.01	2.96
SEC23B	4	2.83
CHAF1B	3.99	4.64
PWP2	3.99	3.93
CGGBP1	3.98	5.7
NUP160	3.98	3.46
PHC2	3.98	4.17
DDOST	3.97	2.65
SNRPA1	3.97	4.51
SRRM1	3.97	2.86
HEL113;VIM	3.96	1.69
BRD7	3.95	2.58
CSTF3	3.95	4.44
HEL-S-103;HSPA1B;HSPA1A	3.95	2.58
RBM33	3.95	3.4
rps2;RPS2;OK/KNS-cl.7	3.95	3.41
SEC61B	3.95	4.81
REPIN1	3.94	4.1
RPL7A;RP-L7a	3.94	3.68
SNRPC	3.94	3.4
YEATS4	3.94	5.29
CCT6A	3.93	3.06
DDX18	3.93	2.7

MED4	3.93	2.66
NSMCE4A	3.93	4.8
SLC25A6	3.93	4.87
CAPZA1	3.92	4.74
CDC16	3.92	4.84
DCAF7	3.92	3.29
MED16	3.92	3.42
NCBP1	3.92	3.55
SEC24C	3.92	4.91
MAP1S	3.91	1.5
SUPV3L1	3.91	2.88
CHEK1	3.9	4.63
SEC13	3.9	3.62
SFRS15;SCAF4	3.89	3.92
SRPRB	3.89	3.91
MACF1	3.88	3.44
NCAPH2	3.88	3.22
EMD	3.87	4.21
INTS7	3.87	6.13
PHC1;DKFZp686A1782	3.87	2.05
RECQL	3.87	3.72
EEF2	3.86	3.63
CUL1	3.85	4.21
SHPRH	3.85	3.34
ZBTB33	3.85	4.13
ZC3H18	3.85	3.05
SUZ12	3.84	2.27
CD2BP2	3.83	4.52
CDC42BPB	3.83	2.09
MORF4L2	3.83	3.91
CSTF2	3.82	2.88
KAT5;HTATIP	3.82	3.13
SAP18	3.82	3.74
HCFC1	3.81	2.07
HNRNPM;ORF	3.81	2.64
LENG8	3.81	4.9
RQCD1	3.81	2.87
TFIP11	3.81	3.89
NHSL1	3.8	2.53
POLE;POLE1	3.8	4.04
SRP68	3.8	2.65
MED10	3.79	2.73
CTBP2	3.77	4.59
IGF2BP1	3.77	4.27
INTS10	3.77	2.79
RCC2	3.77	3.12
DYNC1LI1;DKFZp686A1525	3.76	4.13
EED	3.76	5.26
GTPBP1	3.76	3.88
PRMT3	3.76	3.6

RSBN1L	3.76	4.37
TAF6	3.76	2.17
AAAS	3.75	4.08
GOLGA2	3.75	2.85
SFRS18;PNISR	3.75	5.15
JUN	3.74	2.32
WHSC1	3.74	4.6
INOC1;INO80	3.73	4.36
INTS5	3.71	4.12
NOL6	3.71	2.78
NUP35	3.71	3.76
KPNA1	3.7	4.99
NDNL2	3.7	3.93
TAF9B	3.69	3.23
ZNF592	3.69	2.39
EXO1	3.68	3.75
FLNC	3.67	2.7
GTF2H4	3.67	4.52
NDUFA10	3.67	5.5
EZH2	3.66	2.87
HNRNPA1;HNRPA1;hCG_2020860;RP11-78J21.1;HNRNPA1L2	3.66	2.74
RPS11	3.66	3.62
CCT7	3.65	3.21
DRIP4;PDCD6IP	3.65	2.54
HAUS3	3.64	4.64
LGALS7	3.64	4.04
MED30	3.63	2.61
MTF2	3.62	2.97
NCOR1	3.62	2.14
MYO6	3.61	2.79
NDUFB4	3.61	2.64
NDUFS1	3.61	3.37
TCERG1	3.61	4.72
CIT	3.6	1.24
HEL-S-2a;PRDX2	3.6	3.86
MEN1	3.6	6.6
MEAF6	3.59	2.01
NDC1	3.59	3.2
RP11-98F14.6;PCID2	3.59	3.45
WASF1	3.59	2.95
XRCC6	3.59	1.3
RPL10A	3.58	3.11
FLJ10842;AGK	3.57	3.64
GEMIN4	3.57	2.47
MAU2	3.57	5.13
NFATC1	3.56	5.9
ZNF318	3.56	1.81
CCT3	3.55	3.12

GTF2H2;GTF2H2C	3.55	3.63
HP1BP3	3.55	3.77
UBB;RPS27A;UBC;UBA52;HEL112;DKFZp434K0435;UbC;UBBP4	3.55	3.52
IMPDH;IMPDH1;DKFZp781N0678	3.53	2.84
IQGAP3	3.53	1.69
RALGAPB	3.53	1.76
UBE2L3;hCG_1789329	3.53	2.78
ZC3H14;FLJ11806	3.53	4.19
DARS	3.52	4.88
HIST1H2AB;HIST1H2AC;HIST3H2A	3.52	1.48
SLC25A11	3.52	5.19
KDM2A	3.51	3.78
XPO1	3.51	2.07
HMGXB4	3.5	3.18
PGRMC1	3.5	5.01
POP1	3.5	3.78
RPL18	3.49	3.61
ZNF316	3.49	3.14
CMAS	3.48	9.85
cox2;COX2;COII;MT-CO2	3.48	2.98
VAPA	3.48	3.42
VWA9	3.48	3.54
DUSP11	3.47	3.23
NUDT16L1	3.47	6.74
SNX9	3.47	2.41
ZFC3H1	3.47	1.52
COMMD3-BMI1;BMI1;COMMD3	3.46	3.36
POLR1C	3.46	4.92
AQR	3.45	5.37
DNMT3A	3.45	3.44
SMPD4	3.45	3.32
C10orf12	3.44	2.98
UBL5	3.43	4.04
WDR3	3.43	4.32
BTBD14B;NACC1	3.42	3.14
DNAJA2	3.42	4.05
hCG_2011153;THOC5	3.42	3.33
SNRPN;SNRPB	3.42	4.82
CDK12	3.41	2.27
EXOSC10	3.41	5.82
HSD17B4	3.41	4.81
RBM44	3.41	1.19
EPC2;DKFZP566F2124	3.4	3.01
MED6	3.4	3.41
PHF5A	3.4	3.97

L3MBTL2	3.39	3.56
LIN9	3.39	2.39
NCAPG	3.39	2.79
WDR6;DKFZp434F1720	3.39	5.11
ATP1A1;ATP1A3	3.38	3.54
INTS8	3.38	5.12
PCNT	3.38	3.68
VDAC3	3.37	2.71
ANAPC10;APC10	3.36	3.77
ARID3B	3.36	2.88
CHD3	3.36	4.24
CTCF	3.36	1.83
INTS6	3.36	1.83
NCOR2	3.35	1.73
YARS	3.35	2.4
EHD4	3.34	4.51
AIFM1	3.33	4.12
HEL-S-95n;SORD	3.33	3.01
NR2F2	3.33	3.71
RAVER1	3.33	4.07
TAF5	3.33	2.79
BBX	3.32	2.06
C8orf33	3.32	3.61
CDCA2	3.32	2.8
KHSRP	3.32	1.45
KIF20A	3.32	2.29
NCOA5	3.32	3.19
WDHD1	3.32	4.49
BRCA1	3.31	2.24
FAM48A;SUPT20H	3.3	4.06
THRAP1;MED13	3.3	1.82
TRPS1	3.3	4.68
MLLT10;DKFZp779J0967	3.29	2.96
RPL14	3.29	2.95
MAGI1	3.28	4.94
PAK4	3.28	1.17
MED26	3.27	2.26
NPM3	3.27	3.05
COPB1	3.26	6.96
E4-DBP;hCG_27698;DDX47	3.26	3.27
NKAP	3.26	2.76
RBM25	3.26	1.64
UTP15	3.26	3.47
EDC4	3.25	2.38
MED23	3.25	2.55
PQBP1	3.25	2.56
RBM5	3.25	3.89
RBFOX2	3.24	4.88
ZBTB2;DKFZp666M0210	3.24	2.14
AP2B1;DKFZp781K0743	3.23	2.8

CKAP4	3.23	2.17
PAXBP1	3.23	3.6
USP10	3.23	9.07
HECTD4	3.22	1.71
RBM42	3.22	5.25
DNAJB6	3.21	5.45
XRCC5	3.21	1.16
ASH2L	3.2	2.11
POLR2H	3.2	5.29
SRRT	3.2	5.11
TAF4	3.2	2.02
SNRP70;SNRNP70	3.19	1.01
CDC2L5;CDK13	3.18	3
CREBBP;CBP	3.18	3.17
LUZP1	3.17	2.27
EIF2B4;DKFZp586J0119	3.16	3.95
FAM98A	3.16	3.64
FANCI	3.16	2.73
CDK2	3.15	8.46
MATR3	3.15	2.73
TSG101	3.15	3.44
ANKRD11	3.14	1.7
GEMIN6	3.14	2.94
GPATCH8	3.14	3.04
ERLIN2	3.13	2.71
HADHA	3.13	5.06
IARS;DKFZp686L0869;DKFZp686L17145	3.13	3.38
PPIG	3.13	4.42
SF3B5	3.13	3.95
MED15;DKFZp762B1216;DKFZp686A2214	3.12	2.24
MORC3	3.12	1.8
NOLA1;GAR1	3.12	4.15
RPS4X	3.12	2.75
PPP1R12C	3.11	2.19
CD3EAP	3.1	3.67
KDM1B	3.1	3.37
SLC16A1	3.1	3.35
KPNB1	3.08	2.79
MED31	3.08	3.36
SRGAP2	3.08	1.31
ASCC2;DKFZp586O0223	3.07	2.95
DKFZp686L1814;LIN54;DKFZp686L18142	3.07	1.21
ENY2	3.06	6.17
ABL1;BCR/ABL fusion	3.05	3.7
CDC23	3.05	3.11
TARS2	3.05	3.63
AFF4	3.04	4.03

CDC2L2;CDC2L1;CDK11A;CDK11B	3.04	3.23
LRRC40	3.04	2.75
MYBBP1A	3.04	2.53
SEC11L1;SEC11A;SPC18	3.04	4.39
TADA2B	3.04	4.38
FLNA;FLJ00119	3.03	2.8
INTS4	3.03	7
MAPRE2	3.03	4.37
NPAT	3.03	3.52
PAICS	3.03	1.7
ATAD5	3.02	2.8
NDUFA13	3.02	3.75
THOC1	3.02	5.45
TRAP1	3.02	5.96
URCC5;NFXL1	3.02	3.19
USP7	3.02	3.4
ATM	3.01	2.04
CEBPB	3.01	2.28
EIF3D	3.01	4.48
GLDC	3.01	3.23
LRCH3	3.01	3.35
MSL1	3.01	2.92
NXF1	3.01	3.04
PAWR	3.01	4.09
SEC61A1	3.01	2.8
SLX4	3.01	3.4
hCG_2024613;DNAJC9	3	3.57
IRF2BPL	3	5.39
KTN1	3	3.97
NUP85	3	2.02
CCAR1	2.99	2.73
DARS2	2.99	4.61
HSD17B10	2.98	2.61
LRCH2	2.96	3.75
POLR2C	2.96	1.53
SFSWAP;SFRS8	2.95	3.1
SRP9;DKFZp564M2223	2.95	3.98
ZNF512B	2.95	1.25
CNOT1	2.94	1.22
EIF5B	2.94	6.46
KPNA3	2.94	4.34
SLC25A13	2.94	1.74
HNRNPH1	2.93	2.69
PAPOLG	2.93	5.02
SEC23A	2.92	5.06
CD2AP	2.91	1.58
EIF2S3;EIF2S3L	2.91	4.42
EXOC2	2.91	4.06
ITCH	2.91	4.04

NDUFS2	2.91	2.09
NUP214;DKFZp686J0330	2.91	1.64
PPFIA1	2.91	3.02
TMCO1	2.91	5.35
PTPN13	2.9	1.75
hCG_19665;NUP62	2.89	2.46
KNOP1	2.89	3.34
KRR1	2.89	1.53
PKP2	2.89	4.64
BRCC3	2.88	4.1
CEBPZ	2.88	2.45
POLR2J1;POLR2J;POLR2J3;POLR2J2	2.88	2.27
YTHDC2	2.88	3.08
ACTR3	2.87	3.74
CKAP5	2.87	2.74
POLRMT	2.87	3.91
PRPF31	2.87	3.07
CPSF3L	2.86	2.61
HEL-S-1;YWHAB	2.86	4.8
INTS2	2.86	2.8
FBL	2.85	0.82
KDM3B;JMJD1B	2.85	3.42
NUP54	2.85	3.49
ZNF281	2.84	1.8
CRSP9;MED7	2.83	5.33
HAUS5	2.83	1.88
C8orf36;NSMCE2	2.81	2.1
POLR1A	2.8	5.25
RANGAP1	2.8	2.39
TUBA1C	2.8	4.02
ZNF516	2.8	1.84
feat;METTL13	2.78	3.27
RPL12	2.78	3.24
RPS6	2.78	3.91
TAF5L	2.78	3.24
DOCK1	2.77	2.53
HNRPK;HNRNPK	2.77	3.25
MTA3;tmp_locus_6	2.77	1.64
NUP88	2.77	2.44
CHTOP	2.76	3.77
EEF1D	2.76	2.25
MED13L	2.76	1.6
MNAT1	2.76	2.65
RBM27;POU4F3	2.76	2.42
TBP;TBPL2	2.76	2.62
BTAF1	2.75	2.39
KAT2A	2.75	3.27
AP2A2	2.74	5.79
ELMSAN1;C14orf43	2.74	2.57

HEL-S-29;HEL-211;CKB	2.74	2.26
HEL-S-97n;PRDX4	2.74	7.11
MBIP	2.74	3.82
TUBGCP3	2.74	2.87
NOLC1	2.73	2.63
ZNF384	2.73	3.68
06-Sep	2.72	2.54
AP3D1	2.72	3.71
DDX56	2.7	1.33
SMNDC1	2.7	3.62
CAPZB	2.69	2.98
CTSB	2.69	2.07
NUP133	2.69	1.23
ASNS	2.68	3.19
PLEKHC1;FERMT2	2.68	2.64
TPP1	2.68	3.33
WDR59	2.68	3.63
CCT5;HEL-S-69	2.67	2.91
SAMD1	2.67	4.99
MED9	2.66	2.32
CS	2.65	1.49
NFRKB	2.65	1.95
SGPL1	2.65	3.4
TRIP6;TRIP6i1	2.65	3.85
ZNF292	2.65	1.12
NUP188	2.64	2.27
TUBA1B;TUBA4A	2.63	2.21
INO80E;FLJ00079	2.62	3.41
TGM3	2.62	4.21
PTGES3	2.61	2.14
CYC1	2.6	1.81
CNOT3;FLJ00420	2.59	4.84
EMSY;C11orf30	2.59	1.72
TRIM33	2.59	2.73
UHRF1	2.59	1.12
AKAP8;DKFZp586B1222	2.58	2.04
SRPR	2.58	3.63
CDK2AP1	2.57	2.83
CPNE1	2.57	2.08
NCOA6	2.57	1.18
PARP1	2.57	1.78
PHC3	2.57	2.28
ABI2;ABI-2	2.56	2.4
ATP5C1	2.56	8.11
CENTG3;AGAP3	2.56	3.47
FARSB	2.56	5.1
BOD1L1	2.55	2.02
KLF16	2.55	4.26
CETN2	2.54	5.06
DHRS2	2.54	3.08

RPL3;rpl3	2.54	1.79
WASF2	2.54	1.43
UPF2	2.53	7.32
TMEM33;SHINC3	2.52	3.71
09-Sep	2.51	2.14
HOXA5	2.51	2.34
TFAP4	2.51	2.97
HK2;DKFZp686M1669	2.5	2.94
MCTS1	2.5	3.61
PIAS4	2.5	3.97
ABCD3	2.49	4.84
CUX1	2.49	1.36
SEC63	2.48	1.64
PIK3C2A	2.47	4.34
RAP80;UIMC1	2.47	2.38
TIMM50	2.47	3.21
FAF2	2.46	2.22
HEL-S-89n;HSPA5	2.46	3.21
IQGAP2	2.46	2.27
KCTD15	2.46	1.9
KDM5A	2.46	1.61
RPS6KB2	2.46	3.09
SCRIB	2.46	2.88
MORF4L1	2.45	3.35
MPHOSPH1;KIF20B	2.45	1.44
hCG_2002731;PELO	2.44	3.48
HSP90AB2P	2.44	3.43
MYL12A;MYL12B	2.44	1.96
SEPT5;CDCrel-1	2.44	4.23
CTPS1	2.43	1.73
GEMIN2	2.43	4.5
MPG	2.43	5.97
RBM12B	2.43	1.99
RPL15	2.43	1.85
TRAM1	2.43	4.33
COX4NB;EMC8	2.42	2.88
DKFZp586K0821;NDUFS3	2.42	2.96
KPNA2	2.41	0.74
SEC16A	2.41	3.71
UHRF2	2.41	2.75
BAIAP2	2.4	2.93
KAT6A;MYST3	2.4	1.68
FBRS	2.39	4.02
LRRC47	2.38	4.07
DDX5;DKFZp686J01190	2.37	1.49
DHX36	2.37	4.28
FARSLA;FARSA	2.37	3.29
DDB1	2.36	1.77
ETFA	2.36	2.07
KLC2	2.36	2.15

MCM3AP	2.36	2.37
MARS	2.35	2.02
PUF60	2.35	2.97
TAOK2	2.35	3.32
U2AF2	2.35	1.1
KMT2C;MLL3	2.33	1.23
PELP1	2.33	2.86
TAGLN2	2.33	1.99
CHTF18;RUVBL	2.32	1.55
EIF2AK2	2.32	4.17
HIST2H2AA3;HIST2H2AC	2.32	1.3
RARS	2.32	3.26
SMAD4	2.32	1.95
STT3B	2.32	2.87
KIF18B	2.31	2.74
PALLD	2.31	2.57
PRMT5	2.31	4.44
KIAA1267;KANSL1	2.3	1.17
HEL-S-28;ILK	2.29	1.98
YTHDF2	2.29	5.35
ATP5O	2.28	2.76
ARL6IP4;FLJ00169	2.27	1.98
CDK7	2.27	1.74
HNRNPU;HNRPU	2.27	2.32
SF3B1	2.26	2.83
TFDP1;hCG_1982709	2.26	3.83
NDUFA9	2.25	3
CEP170B	2.24	1.48
PRKDC	2.24	2.53
EIF2B3	2.22	2.65
DKFZp762M013;CRNKL1;crn	2.21	2.55
hCG_17415;SLAIN2	2.21	1.53
VPS37B	2.2	4.8
HSPA14	2.19	4.83
TRIP12	2.19	2.02
ABL2	2.18	1.18
CFL1;HEL-S-15	2.18	1.23
TBL1X;TBL1Y	2.18	2.44
NELFB	2.17	2.42
MYH10	2.15	5
PCGF6	2.15	1.34
ETF1	2.14	4.26
TAF1;TAF1L	2.14	1.75
SMAD2;SMAD3;SMAD9	2.13	5.34
ATR	2.12	3.58
PREB	2.12	1.83
SLC25A22	2.11	3.3
SPATA5	2.11	2.79
CCT8	2.1	3.71
EXOSC4	2.1	4.05

NAA40;NAT11	2.1	2.66
ABCF1	2.09	7.01
COX7A2	2.08	1.55
EEF1A2	2.06	4.97
KMT2B	2.06	1.09
SF3B4	2.05	1.91
HIST1H4H;HIST1H4A	2.04	2.12
EDC3	2.03	2.73
MORC4	2.03	1.51
SUPT3H	2.03	2.58
NOM1	2.02	2.07
CASP14	2.01	3.79
NSUN5	2.01	1.87
TET1	2	1.4
CCT2;HEL-S-100n	1.99	2.63
DKFZp686B2325;DKFZp686L2367;ZNF462	1.99	4.08
ZKSCAN1	1.99	3.72
C7orf26	1.98	2.32
TAF9	1.98	1.75
ACAD11	1.97	1.09
PRPF38B	1.97	7.73
GART	1.96	3.52
MSH6;GTBP	1.96	2.1
PFKL	1.96	1.34
PHF10	1.96	0.98
GLI3	1.95	2.49
MCRS1	1.95	3.87
CTNNA1	1.94	1.44
DIAPH3	1.93	2.62
NOC4L	1.93	2.66
DPM1	1.92	1.89
MYO1B	1.92	1.34
ZFP64	1.92	5.59
S100A2	1.91	2.11
ELMO2	1.9	3.31
HSPA14	1.9	4.05
MTCH2	1.9	1.62
ALDH1B1	1.89	4.49
CLINT1	1.89	2.91
PIAS1	1.89	4.16
TRIM27	1.89	1.77
TMPO	1.86	1.44
ICE1	1.85	1.82
ATP2A2	1.84	5.89
hCG_2039588;PBX2	1.84	2.16
DYNC1LI2	1.83	2.13
CCNA2	1.82	2.75
PPP2R2A;PPP2R2D	1.82	2.53

VCP;HEL-S-70;DKFZp434K0126	1.82	2.12
CCNL1	1.81	2.25
ILF2	1.8	1.1
MEPCE	1.8	1.91
PKN3	1.8	4.21
MPP5	1.79	0.82
NOP56	1.79	2.21
SSR3	1.78	1.59
TK1	1.78	1.76
CAMSAP3;hCG_2004001	1.77	3.33
DOCK11	1.76	1.2
HOMEZ	1.76	1.34
MRI1	1.76	3.86
CC2D1A	1.74	2.99
GMPS	1.74	4.56
ZNF148	1.72	2.29
POLR1B	1.7	3.47
GTF2E1	1.66	3.31
SAMHD1	1.66	3.31
WHSC1L1	1.66	1.31
DKFZp686E1893;NOP9	1.65	2.81
EHD1;EHD3	1.64	2.46
DHX40	1.63	3.82
APEX1	1.62	2.59
EIF3A;eIF3a	1.62	3.25
GTF2H1	1.62	1.73
DKFZp686M05161;TJP1;DKFZp686A1195	1.59	2.45
PLS3	1.59	1.17
RPS10;RPS10-NUDT3	1.59	4.12
DDX23	1.58	3.88
ESPL1	1.57	1.12
EL52;HSP90AA1	1.53	1.28
POLR3A	1.53	1.57
ACADM;DKFZp686M24262	1.52	2.08
VARS	1.52	1.1
CARM1	1.5	4.85
UNC84B;SUN2	1.5	1.28
MTG1	1.48	2.8
MOGS;GCS1	1.47	2.03
BRE	1.45	1.01
DNAJC13	1.45	3.07
EEF1E1;EEF1E1-BLOC1S5	1.43	3.08
ESF1	1.43	2.95
TFAP2A	1.43	3.37
DLAT	1.42	2.76
LANCL2	1.42	2.36
TIMELESS	1.41	2.6
DYNLT3	1.4	0.96

RPP30	1.4	2.38
ATF7	1.39	2.14
DHX38	1.39	2.52
POLD1	1.38	4.14
UQCRQ	1.38	1.38
DKFZp686E2459;RBM10	1.36	3.63
PI4KA;PIK4CA variant protein;hCG_1999854	1.34	1.34
AURKA	1.32	2.58
KIAA1522	1.32	4.03
RAD23B	1.32	1.06
SPFH1;ERLIN1	1.32	1.02
DCAKD	1.3	3.15
FAM60A	1.3	1
BAP1	1.28	2.99
PBX1	1.28	0.83
ZNF740	1.28	3.08
COPE	1.27	4.21
INADL	1.26	5.48
VPS51	1.26	1.92
SIN3B	1.25	0.83
TNRC15;GIGYF2	1.25	1.94
ERCC4	1.24	1.35
ABCE1	1.23	2.75
SLC25A10	1.23	2.82
ZC3H4	1.22	0.8
HUWE1	1.21	1.66
LRRC41	1.21	0.99
IQSEC1	1.2	0.92
GPATCH4	1.19	2.41
YARS2	1.18	1.25
MKRN2	1.17	1.55
CDC40	1.13	1.72
PRKAR2A	1.12	2.01
DYNC2LI1	1.08	1.79
OSBPL8;DKFZp686C0249	1.08	1.14
SCMH1	1.03	1.39
DHX8	1.02	0.76
DDX21	1	0.71
EIF4G2;AAG1	0.99	2.68
FBXL18	0.99	1.27
NCAPH	0.99	1.08
GLRX3	0.97	3.5
HDAC3	0.97	2.77
PRDX1	0.96	0.93
LRPPRC	0.95	2.47
OAT	0.93	3.82
POLA2	0.91	2.66
DRG1	0.9	6.9
FOXF2	0.88	3.2

VPS33B	0.86	5.96
FMR1	0.85	0.79
TLN1	0.85	1.26
IPO4	0.84	1.88
PIGT	0.84	2.14
SMC3	0.84	1.2
ERCC6L	0.83	1.69
FAM62A;ESYT1	0.83	1.97
STK26;MST4;STK25;DKFZp686J1430	0.81	1.31
XPOT	0.81	1.25
JMJD1C	0.77	1.37
EXOC7;DKFZp686P1551	0.75	0.84
NOL11	0.72	2.1
RPRC1;MAP7D1	0.68	1.31
GPATCH1	0.66	1
MTMR1	0.65	1.22
MRPL10	0.63	0.94
SYNE2;TROPH	0.63	3.15
URB2	0.53	0.71
SERPINH1	0.52	3.99
ACTG1	0.47	0.72
GSK3B	0.46	0.94
XPO5	0.45	0.69
PSMC4	0.42	1.46
EIF2B1	0.36	2.22
ACAT1	0.28	1.18
NAA25	0.13	1.05
