# Supplementary material for: ARID1A regulates DNA repair through chromatin organization and its deficiency triggers DNA damage-mediated anti-tumor immune response
Source: Nucleic Acids Res. 2024 Apr 8;52(10):5698–719. doi: 10.1093/nar/gkae233 (PMC11162808; doi:10.1093/nar/gkae233)
Supplement: gkae233_Supplemental_Files [file gkae233_supplemental_files.zip › Supplementary Table S4_DSB repair-related factors.pdf]

Supplementary Table S4: list of the identified DNA repair related proteins

names	log2FoldChange	FDR pvalue (-log10)
CHD4	9.13	2.04
SFPQ	9.12	5.67
MSH2	7.49	8.99
POGZ	7.48	5.83
TP53BP1	7.4	5.39
SMCHD1	7.19	6.1
RAD50	7.15	7.39
PRPF19	7.13	4.21
MCM7	7.07	5.05
NIPBL	6.89	5.86
MCM4	6.58	5.17
DDX1	6.05	5.26
MCM6	6.05	4.89
MRE11	5.9	5.02
MCM5	5.84	6.71
MCM2	5.4	5.29
SMC5	5.14	5.02
MCM3	5.04	5.73
TNKS1BP1	4.9	2.39
INTS3	4.88	4.9
NBN	4.66	5.27
RAD21	4.64	5.64
SMC6	4.58	3.9
BLM	4.3	3
NSMCE4A	3.93	4.8
RECQL	3.87	3.72
KAT5	3.82	3.13
KDM2A	3.51	3.78
BRCA1	3.31	2.24
SLX4	3.01	3.4
ATM	3.01	2.04
BRCC3	2.88	4.1
NSMCE2	2.81	2.1
INO80	2.62	3.41
PARP1	2.57	1.78
PIAS4	2.5	3.97
UIMC1	2.47	2.38
MORF4L1	2.45	3.35
PRKDC	2.24	2.53
